# DNA methylation patterns associate with genetic and gene expression variation in HapMap cell lines

**DOI:** 10.1186/gb-2011-12-1-r10

**Published:** 2011-01-20

**Authors:** Jordana T Bell, Athma A Pai, Joseph K Pickrell, Daniel J Gaffney, Roger Pique-Regi, Jacob F Degner, Yoav Gilad, Jonathan K Pritchard

**Affiliations:** 1 Department of Human Genetics, The University of Chicago, 920 E. 58th St, Chicago, IL 60637, USA; 2 Howard Hughes Medical Institute, The University of Chicago, 920 E. 58th St, Chicago, IL 60637, USA; 3 Wellcome Trust Centre for Human Genetics, University of Oxford, Roosevelt Drive, Oxford OX3 7BN, UK

## Abstract

**Background:**

DNA methylation is an essential epigenetic mechanism involved in gene regulation and disease, but little is known about the mechanisms underlying inter-individual variation in methylation profiles. Here we measured methylation levels at 22,290 CpG dinucleotides in lymphoblastoid cell lines from 77 HapMap Yoruba individuals, for which genome-wide gene expression and genotype data were also available.

**Results:**

Association analyses of methylation levels with more than three million common single nucleotide polymorphisms (SNPs) identified 180 CpG-sites in 173 genes that were associated with nearby SNPs (putatively in *cis*, usually within 5 kb) at a false discovery rate of 10%. The most intriguing *trans *signal was obtained for SNP rs10876043 in the disco-interacting protein 2 homolog B gene (*DIP2B*, previously postulated to play a role in DNA methylation), that had a genome-wide significant association with the first principal component of patterns of methylation; however, we found only modest signal of *trans*-acting associations overall. As expected, we found significant negative correlations between promoter methylation and gene expression levels measured by RNA-sequencing across genes. Finally, there was a significant overlap of SNPs that were associated with both methylation and gene expression levels.

**Conclusions:**

Our results demonstrate a strong genetic component to inter-individual variation in DNA methylation profiles. Furthermore, there was an enrichment of SNPs that affect both methylation and gene expression, providing evidence for shared mechanisms in a fraction of genes.

## Background

DNA methylation plays an important regulatory role in eukaryotic genomes. Alterations in methylation can affect transcription and phenotypic variation [1], but the source of variation in DNA methylation itself remains poorly understood. Substantial evidence of inter-individual variation in DNA methylation exists with age [2,3], tissue [4,5], and species [6]. In mammals, DNA methylation is mediated by DNA methyltransferases (DNMTs) that are responsible for de novo methylation and maintenance of methylation patterns during replication. Genes involved in the synthesis of methylation and in DNA demethylation can also affect methylation variation. For example, mutations in DNMT3L [7] and MTHFR [8] associate with global DNA hypo-methylation in human blood. These changes occur at a genome-wide level and are distinct from genetic variants that impact DNA methylation variability in targeted genomic regions, for example, genetic polymorphisms associated with differential methylation in the *H19/IGF2 *locus [9].

Recent evidence suggests a dependence of DNA methylation on local sequence content [10-12]. A strong genetic effect is supported by studies of methylation patterns in families [13] and in twins [14], but stochastic and environmental factors are also likely to play an important role [2,14]. Recent work indicates that genetic variation may have a substantial impact on local methylation patterns [5,15-18], but neither the extent to which methylation is affected by genetic variation, nor the mechanisms are yet clear. Furthermore, the degree to which variation in DNA methylation underlies variation in gene expression across individuals remains unknown.

DNA methylation has long been considered a key regulator of gene expression. The genetic basis of gene expression has been investigated across tissues [19] and populations [20]. Both lines of evidence suggest genetic variants associated with gene expression variation are located predominantly near transcription start sites. However, not much is known about the precise mechanisms by which genetic variants modify gene-expression. Combining genetic, epigenetic, and gene expression data can inform the underlying relationship between these processes, but such studies are rare on a genome-wide scale. Two recent studies have examined the link between DNA methylation and expression in human brain samples [5,18]. Both studies identified substantial numbers of quantitative trait loci underlying each type of phenotype, but few examples of individual loci driving variation in both methylation and expression.

To better understand the role of genetic variation in controlling DNA methylation variation, and its resulting effects on gene expression variation, we studied DNA promoter methylation across the genome in 77 human lymphoblastoid cell lines (LCLs) from the HapMap collection. These cell lines represent a unique resource as they have been densely genotyped by the HapMap Project [21], and are now being genome-sequenced by the 1,000 Genomes Project. In addition, these cell lines have been studied by numerous groups studying variation in gene expression using microarrays [20,22] and RNA sequencing [23,24], as well as smaller studies of variation in chromatin accessibility and PolII binding [25,26]. Finally, one of the HapMap cell lines is now being intensely studied by the ENCODE Project [27]. This convergence of diverse types of genome-wide data from the same cell lines should ultimately enable a clearer understanding of the mechanisms by which genetic variation impacts gene regulation.

## Results

### Characteristics of DNA promoter methylation patterns

To study inter-individual variation in methylation profiles we measured methylation levels across the genome in 77 lymphoblastoid cell lines (LCLs) derived from unrelated individuals from the HapMap Yoruba (YRI) collection. For these samples we also had publicly available genotypes [21], as well as estimates of gene expression levels from RNA-sequencing in 69 of the 77 samples [24]. Methylation profiling was performed in duplicate using the Illumina HumanMethylation27 DNA Analysis BeadChip assay, which is based on genotyping of bisulfite-converted genomic DNA at individual CpG-sites to provide a quantitative measure of DNA methylation. The Illumina array includes probes that target 27,578 CpG-sites. However, we limited analyses to probes that mapped uniquely to the genome and did not contain known sequence variation, leaving us with a data set of 22,290 CpG-sites in the promoter regions of 13,236 genes (see Methods). Following hybridization, methylation levels were estimated as the ratio of intensity signal obtained from the methylated allele over the sum of methylated and unmethylated allele intensity signals. Methylation levels were quantile-normalized [28] across two replicates. We tested for correlations with potential confounding variables that could affect methylation levels in LCLs [29], such as LCL cell growth rate, copy numbers of Epstein-Barr virus, and other measures of biological variation (see Additional file 1) that were available for 60 of the individuals in our study [30]; these did not significantly explain variation in the methylation levels in our sample (Figure S1 in Additional file 1). However, we observed an influence of HapMap Phase (samples from Phase 1/2 vs 3) on the distribution of the first principal component loadings in the autosomal data, suggesting that the first methylation principal component may in part capture technical variation potentially related to LCL culture. In the downstream association mapping analyses, we applied a correction using principal component analysis regressing the first three principal components to account for unmeasured confounders and increase power to detect quantitative trait loci.

### Global patterns of methylation

**Figure 1:**
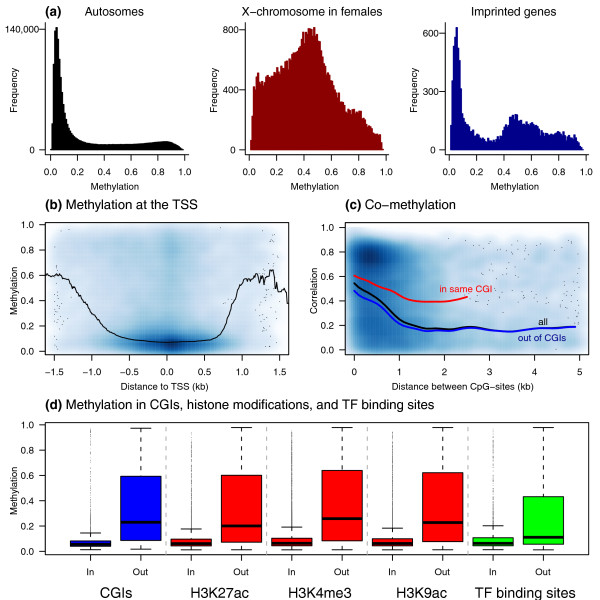
**Distribution of methylation patterns across the genome**. **(a) **Methylation patterns for CpG-sites on autosomes, X-chromosome, and in the vicinity of imprinted genes. Methylation values are plotted for 77 individuals at 21,289 autosomal CpG-sites (left), for 43 females at 997 CpG-sites on the X-chromosome (middle), and for 77 individuals at 153 CpG-sites in 33 imprinted genes (right). **(b) **Methylation levels with respect to the TSS (negative distances are upstream from the TSS), where the line represents running median levels in sliding windows of 300 bp. **(c) **Correlations in methylation levels for all pair-wise CpG-sites (black), and for CpG-sites where both probes are in the same CGI (red), or where at least one probe is outside of CGIs (blue). Lines indicate smoothed spline fits of the mean rank pairwise correlation between CpG-sites in 100 bp windows, weighted by the number of probe pairs. **(d) **Methylation levels inside and outside of annotation categories, including CpG Islands (CGIs) for probes within 100 bp of the TSS, and histone modifications and transcription factor (TF) binding sites for all probes (see Additional file 1).

Distinct patterns of methylation were observed for CpG-sites located on the autosomes, X-chromosome, and in the vicinity of imprinted genes (Figure 1a). The majority (71.4%) of autosomal CpG-sites were primarily unmethylated (observed fraction of methylation <0.3), 15.6% were hemi-methylated (fraction of methylation was between 0.3 and 0.7), and 13% were methylated. As expected, these patterns were consistent with previously observed lower levels of methylation near promoters relative to genome-wide levels [4,31]. We did not find evidence for sex-specific autosomal methylation patterns, consistent with a previous report [4]. In contrast, CpG-sites on the X-chromosome exhibited highly significant sex-specific differences (Figure S2) with hemi-methylated patterns in females that were consistent with X-chromosome inactivation. A similar hemi-methylation peak was observed for CpG-sites located near the transcription start sites (TSSs) of known autosomal imprinted genes in the entire sample.

We observed a previously reported [4] drop in methylation levels for CpG-sites located within 1 kb of TSSs (Figure 1b). Promoter methylation levels have been reported to vary with respect to CpG islands [32]. We found that although distance to the CpG island (CGI) border [33] (including CpG shores [34]) did not significantly affect methylation levels, CpG-sites located in CGIs were under-methylated and less variable (Wilcoxon rank-sum test *P *< 2.2 × 10^-16^) compared to sites outside of CGIs (Figure 1, Figure S3 in Additional file 1).

Methylation is often found to be correlated across genomic regions at the scale of 1-2 kb [4,35]. We investigated whether the correlation between autosomal methylation levels (co-methylation) depended on the distance between CpG-sites. We observed that methylation levels at probes located in close proximity (up to 2 kb apart) were highly correlated (Figure 1c), indicating that variation in methylation levels between individuals is correlated within cell type. Figure 1c also shows that pairs of CpG-sites that were both within a CGI showed greater evidence for co-methylation than pairs of CpG sites for which at least one was outside the CGI, controlling for distance, implying differential regulation of DNA methylation for CpGs inside and outside of CGIs [32].

### DNA methylation correlates with transcription and histone modifications

**Figure 2:**
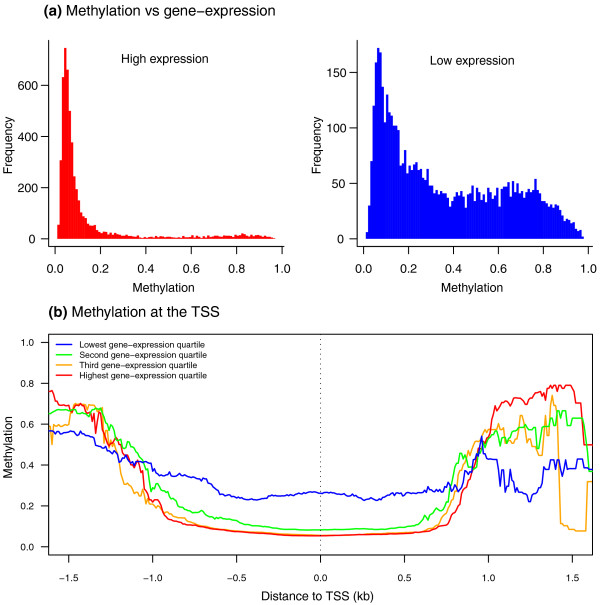
**DNA methylation is negatively correlated with gene expression**. **(a) **Methylation levels are low in the top quartile of highly expressed genes (left), and high in the bottom quartile of lowly expressed genes (right), looking across 12,670 autosomal genes. **(b) **Methylation levels with respect to the TSS in sets of genes categorized by gene expression levels, from highest (red) to lowest (blue), using the quartiles of gene expression with respect to gene expression means, where fitted lines represent running median levels (see Figure 1b).

Methylation has long been implicated in the regulation of gene expression. To examine the role of methylation in gene expression variation, we compared methylation levels to estimates of gene expression based on RNA-sequencing (Figure 2a). Within individuals, we found a significant negative correlation between methylation and gene expression levels (Figure S4 in Additional file 1) across 11,657 genes (mean rank correlation *r *= -0.454). We divided the genes into quartiles from high to low gene expression and observed that the drop in methylation levels near to the TSS (Figure 1b) was only seen in highly expressed genes (Figure 2b). We also asked whether variation in methylation levels across individuals correlates with variation in gene expression levels. Comparisons at the gene level across 69 individuals indicated a modest but significant excess of negatively correlated genes (permutation *P *< 0.0001).

DNA methylation is thought to interact with histone modifications during the regulation of gene-expression [36,37]. We compared methylation levels in our sample with histone modification ChIP-seq data from the ENCODE project in one of the CEPH HapMap LCLs (GM12878). We found strong negative correlations between DNA methylation levels and the presence of histone marks that target active genes (Figure 1d; Figures S3 and S5 in Additional file 1). For example, DNA methylation was low in H3K27ac peaks, which are indicative of enhancers [38], have previously been positively correlated with transcription levels [39] and negatively correlated with DNA methylation levels [31]. Similarly, the transcription marks H3K4me3 and H3K9ac were both negatively correlated with DNA methylation levels. We also observed lower methylation levels in transcription factor binding sites predicted by the CENTIPEDE algorithm, using cell-type specific data including DNase1 sequencing reads [40], consistent with the expectation that the absence of methylation is important for transcription factor binding.

### Genome-wide association of DNA methylation with SNP genotypes

We next assessed whether genetic variation contributes to inter-individual variation in DNA methylation levels. We first tested whether any SNPs were associated with overall patterns of DNA methylation, as measured by principal component analysis (see Methods). The most interesting signal was obtained for SNP rs10876043, which had a genome-wide significant association with variation in the first principal component of methylation (*P *= 4.5 × 10^-9^), and which also showed a modest association with average genome-wide methylation levels (*P *= 4.0 × 10^-5^) (Table S1 in Additional file 1). This SNP lies within the intron of the gene *DIP2B*, which contains a DMAP1-binding domain, and has been previously proposed to play a role in DNA methylation [41].

#### Associations in trans

After assessing the possibility that SNPs can have genome-wide effects on overall methylation patterns, we next transformed the methylation data by regressing out the first three principal components (see Methods), as we have previously found that this procedure can greatly reduce noise in the data and improve quantitative trait locus (QTL) mapping [24] (see also [42,43]). At a genome-wide false discovery rate (FDR) of 10% (*P *= 2.1 × 10^-10^) methylation levels at 37 CpG-sites showed evidence for association with SNP genotypes (Table S2 in Additional file 1). The majority of these CpG-sites (27 of 37) were putative *cis *association signals, that is, the most significant SNP was within 50 kb of the measured CpG site (Figure S6 in Additional file 1). We observed a modest enrichment of distal associations (putative *trans *associations) that was primarily due to signals in 10 CpG-sites (Figure S7 in Additional file 1). We then examined distal association at SNPs that had previously been implicated in methylation (Table S3 in Additional file 1) and found a significant proximal association between SNP rs8075575, which is 150 kb from gene *ZBTB4 *that binds methylated DNA, and methylation at probe cg24181591 in gene *EIF5A *that encodes a translation initiation factor. Three previously reported [5] significant distal associations were also observed for SNP rs7225527 (38 kb from gene *RHBDL3*) and methylation at probe cg17704839 in gene *UBL5 *that encodes ubiquitin-like protein, and for SNPs rs2638971 (106 kb from gene *DDX11*) and rs17804971 (49 kb from gene *DDX12*) and methylation at probe cg18906795 in gene *RANBP6*, which may function in nuclear protein import as a nuclear transport receptor. Associations were also seen at SNPs located 165 kb from the gene encoding methyl-binding protein *MBD2*, 22 kb from the methyltransferase gene *DNMT1*, 192 kb from the methyltransferase gene *DNMT3B*, and at three SNPs with previous evidence for association but to different regions [16] (Figure S8 in Additional file 1). Overall however, we obtained relatively weak evidence for associations in *trans *and weak to moderate enrichment of *trans *association signals at more relaxed significance thresholds in candidate regions of interest.

#### Associations in cis

**Figure 3:**
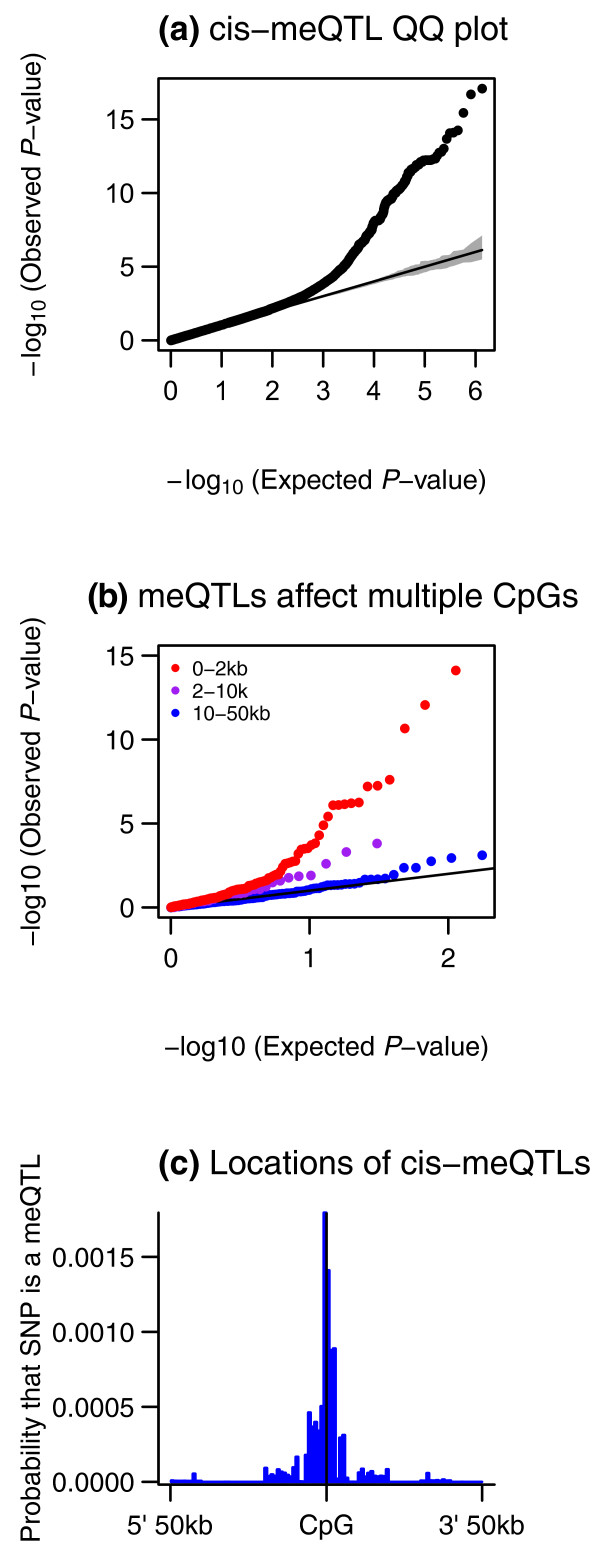
***Cis *methylation QTLs**. **(a) **Quantile-quantile (QQ) plot describing the enrichment of association signal in *cis *compared to the permuted data (90% confidence band shaded). **(b) **The *cis*-meQTL SNPs were enriched for association signal at additional CpG-sites near to the CpG-site for which they are meQTLs. The 180 best-associated SNPs were tested for association to probes that fell within 2 kb (red), within 2 kb to 10 kb (purple), and within 10 kb to 50 kb (blue) of the original best-associated CpG-site. The majority (96%) of probes within 2 kb (red) were in the same CGI as the best-associated probe. **(c) **Spatial distribution of *cis*-meQTLs with respect to the CpG-site as estimated by the hierarchical model.

Since the majority of the genome-wide association signals were proximal to the corresponding CpG-sites, we next focused on association testing for SNPs within 50 kb of each CpG-site (Figure 3). At a genome-wide FDR of 10% (*P *= 2.0 × 10^-5^) there were 180 CpG-sites with *cis *methylation quantitative trait loci (meQTLs). The strongest association signal (*P *= 8.0 × 10^-18^) was obtained at SNP rs2187102 with probe cg27519424 in gene *HLCS*, which is thought to be involved in gene-regulation by mediating histone biotinylation [44]. The proportion of variance explained by meQTLs for normalized methylation data ranged between 22% and 63%. If mechanisms affecting DNA methylation generally act over distances of up to approximately 2 kb (Figure 1c), then SNPs impacting methylation should be detected as meQTLs at multiple nearby CpG-sites. We observed that SNPs associated with methylation were also enriched for association with additional CpG-sites within 2 kb of the best-associated CpG-site with the most-significant *P-*value (Figure 3b), suggesting that a single genetic variant often affects methylation at numerous nearby CpG-sites.

Genetic variation has previously been associated with methylation at specific imprinted regions [1]. The 180 CpG-sites with meQTLs in our data were nearest to the TSSs of 173 genes, of which two-*MEST *and *CPA4*, were known to be imprinted genes. Previous observations suggested that eQTL and imprinting effects can be sex-specific [45], raising the possibility that some of the meQTLs may act in a sex-dependent manner. However, we did not find compelling genome-wide significant sex-specific *cis *meQTL effects (see Additional file 1). Of the 180 associations of CpG-sites with proximal meQTLs, 27 were previously reported in human brain samples [5].

Little is known about the biological mechanisms that may underlie meQTL effects. To this end we applied a Bayesian hierarchical model [22] to test for enrichment of meQTLs in transcription factor binding sites, in histone modification categories, and in the vicinity of the associated probes. We found that SNPs located nearest to the probe, and specifically in the 5 kb immediately surrounding the probe, were significantly enriched for meQTLs (Figure 3c). Transcription factor binding sites, including CTCF-binding sites, showed a modest but non-significant enrichment for meQTLs (Figure S9 in Additional file 1).

### Methylation QTLs are enriched for expression QTLs

**Figure 4:**
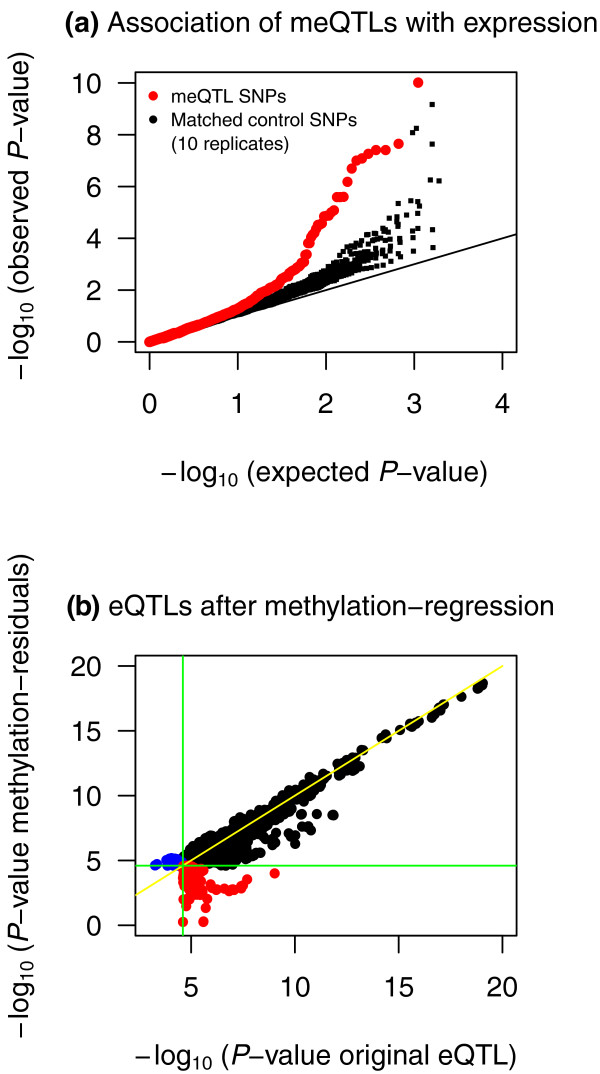
**The overlap between meQTLs and eQTLs**. **(a) **QQ-plot describing the eQTL association *P-*values in 180 *cis*-meQTL SNPs (red) and in eight samples of SNPs that match the *cis*-meQTL SNPs for minor allele frequency and distance-to-probe distributions (black). **(b) **Association signals in 508 FDR 10% eQTLs before and after regressing out gene-specific methylation. In black are 439 eQTLs that overlap across the two phenotypes, in red are 45 eQTLs present before methylation regressions, and in blue are 24 eQTLs present after regressing out methylation. The flat lines (green) correspond to the FDR 10% eQTL threshold.

Finally, we examined the overlap in regulatory variation that affects both methylation and gene expression levels using RNA-sequencing data [24]. We hypothesized that since DNA methylation can regulate gene expression, then variants that affect methylation should often have consequent effects on gene expression. The first way that we looked at this was to take the set of 180 SNPs that are meQTLs at FDR <10% (taking only the most significant SNP for each meQTL). We then tested each of these SNPs for association with expression levels of nearby genes (Figure 4a, red points). There is a clear enrichment of association with expression levels compared to the null hypothesis (black line) and compared to sets of control SNPs that are matched in terms of allele frequency and distance-to-probe distributions (black dots).

**Figure 5:**
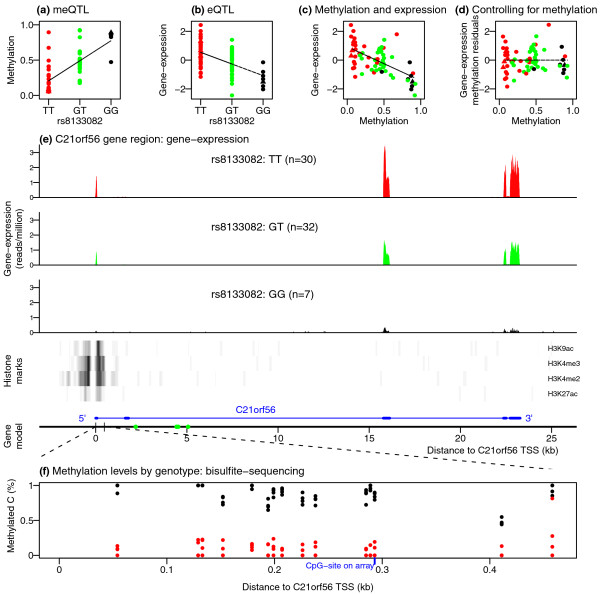
***C21orf56 *gene region**. **(a)**, **(b)**, **(c) **Genotype at rs8133082 is associated with methylation (cg07747299) and gene expression at *C21orf56*, plotted per individual colored according to genotype at rs8133082 (GG = black, GT = green, TT = red) for directly genotyped (circles) and imputed (triangles) data. **(d) **Gene expression levels at *C21orf56 *after regressing out methylation. **(e) **Gene expression at *C21orf56 *(+/-2 kb) genomic region on chromosome 21. Distance is measured on the reverse strand relative to *C21orf56 *TSS at 46,428,697 bp. Barplots show average gene expression reads per million in the subsets of individuals from each of the three rs8133082-genotype classes. Middle panel shows histone-modification peaks in the region from Encode LCL GM12878. Bottom panel shows the gene-structure of *C21orf56*, where exons are in bold and the gene is expressed from the reverse strand. Green points indicate the location of four HapMap SNPs (rs8133205, rs6518275, rs8133082, and rs8134519) associated at FDR of 10% with both methylation and gene expression, and Figure S11 in Additional file 1 shows association results for this region with SNPs from the 1,000 Genomes Project. **(f) **Bisulphite-sequencing results for eight rs8133082-homozygote individuals (4 GG black, 4 TT red) validates the genome-wide methylation assay at cg07747299 and shows the extent of methylation in the surrounding 411 bp region.

One example of a SNP, rs8133082, that is both a meQTL and eQTL for the gene *C21orf56 *is illustrated in Figure 5. When we regress out methylation, this completely removes the association of this SNP with gene expression (Figure 5a, b, c, d). We validated the methylation assay findings at *C21orf56 *by bisulfite sequencing the methylation probe region in eight samples in our study, four from each homozygote genotype class for the SNP (Figure 5f). The two methylation probes at *C21orf56 *both had cis meQTLs and overlapped the likely promoter region as indicated by histone modification data (Figure 5e), suggesting that genetic variation may affect the chromatin structure in this region. *C21orf56 *appears to modulate the response of human LCLs to alkylating agents, and may act as a genomic predictor for inter-individual differences in response to DNA damaging agents [46].

To examine further the overlap between eQTLs and meQTLs, we re-analyzed the eQTL data by incorporating methylation as a gene-specific covariate. If variation in methylation underlies variation in gene-expression, we expect to observe a drop in the number of eQTLs in the methylation-residual gene expression data. At an FDR of 10% (*P *= 2.5 × 10^-5^) there were 484 original eQTLs and 463 methylation-residual eQTLs, where 439 eQTLs overlapped, 45 eQTLs were present only in the original data, and 24 new eQTLs were present only in the methylation-residuals (Figure 4b). Interestingly, the SNPs that were eQTLs for the 45 genes with reduced signals in the methylation-residuals were enriched for significant methylation associations (Figure S10 in Additional file 1), suggesting that these are true underlying meQTLs, where genetic variation affects methylation, which in turn regulates gene expression [5,18]. In summary our results indicate a significant enrichment of SNPs that affect both methylation and gene expression, suggesting a shared mechanism (for example, that increased DNA methylation might drive lower gene expression). However the number of genes that show such a signal is a modest fraction of the total number of meQTLs.

## Discussion

We report association between DNA methylation with genetic and gene expression variation at a genome-wide level. We have identified methylation QTLs genome-wide, the majority of which act over very short distances, namely less than 5 kb. Furthermore, methylation patterns generally covary within individuals over distances of approximately 2 kb and in conjunction with this, meQTLs frequently affect multiple neighboring CpG sites. Our findings are consistent with previous methylation associations [5,16,18], familial aggregation [13,14], correlation with local sequence [10], allele-specific methylation [15,17], and effects of histone modifications [47]. Little is known about the biological mechanisms that underlie meQTL effects, however, this is one important route to identify how genetic variation affects gene regulation.

We find an overall enrichment of significant associations of genetic variants with methylation CpG-sites, which is consistent with the results from two recent reports examining genome-wide methylation QTLs in human brain samples [5,18]. Overall, the number of genome-wide significant meQTLs varies across the three studies, which is likely due to differences in sample sizes, differences in multiple testing corrections and definition of *cis *intervals, and the presence of large tissue-specific differences in DNA methylation with tissue-specific meQTLs. In general, power to detect meQTLs will depend on many factors including sample size, genome-wide coverage of genetic variation, genome-wide coverage of methylation variation, and the effect size of the genetic variants associated with methylation variation in the tissue of interest.

Additionally, our analyses are based on Epstein-Barr virus transformed lymphoblastoid cell lines. The choice of cell type will affect the observed genome-wide DNA methylation patterns, and in particular, high-passage LCLs may exhibit methylation alterations over time [29]. Sun *et al*. [48], for example, investigated genome-wide differences in DNA methylation between LCLs and peripheral blood cells (PBCs), and identified 3,723 autosomal DNA methylation sites that had significantly different methylation patterns across cell types. In that respect, it is expected that a subset of our results reflect LCL-specific events. We have tested potential confounding variables that could affect methylation levels specifically in LCLs [30], but do not observe significant effects of these on overall DNA methylation patterns in our data. However, variation in methylation are slightly different in HapMap Phase 1/2 samples compared to HapMap Phase 3 samples, suggesting that technical variation related to LCL culture may influence DNA methylation. We took this into account when performing all downstream methylation QTL analyses, and our analyses of the uncorrected methylation patterns are consistent with the results of previous studies in primary cells [4,31,35].

We obtained interesting results from the *trans *analysis highlighting several loci with potential long-range effects on DNA methylation. Furthermore, an intriguing association of a SNP within the intron of *DIP2B*, which contains a DMAP1-binding domain, with the first principal component of autosomal methylation patterns suggests novel genome-wide effects on methylation variability. However, we do not observe a strong effect of polymorphisms in many of the candidate methylation regulatory genes on overall patterns of methylation or on specific probes. The sample size used in the study limits our power to detect *trans *signals, rendering these analyses more difficult to interpret. In general, the moderate sample sizes used in all three genome-wide methylation studies to date do not allow for the detection of subtle effects of genetic variants on methylation variation and correspondingly the majority of methylation sites assayed across all studies remains unexplained by the GWAS analyses. However, the findings indicate that genetic regulation of methylation is as complex as expression or phenotypic variation.

Relating genetic variation to both DNA methylation and gene expression variation reveals complex patterns. We observe significant overlap between meQTLs and eQTLs for *cis *regulatory variants. These findings were obtained when we both focus exclusively on meQTL SNPs (Figure 4a) and when we compare the genome-wide meQTL results for all SNPs classified as eQTLs in the hierarchical model framework (Figure S9 in Additional file 1). The observations indicate evidence for shared regulatory mechanisms in a fraction of genes. However, in the re-analyses of the eQTL data taking into account DNA methylation, in only 10% of eQTLs was the genetic effect of the SNP on expression affected by controlling for methylation, suggesting that variation in methylation accounts for only a small fraction of variation in gene expression levels. There may be several explanation for this. First, the coverage of the methylation array provides a relatively low resolution snapshot of the genome-wide DNA methylation patterns. Second, steady state gene expression levels (as measured by RNA-sequencing) are controlled by many other factors in addition to DNA methylation, such as transcription factor binding, chromatin state including histone marks and nucleosome positioning, and regulation by small RNAs. Finally, our study sample size provides modest power, both for eQTL and meQTL mapping. However, compared to previous studies addressing this issue [5,18], we find more convincing evidence for meQTL and eQTL overlap. For example, Zhang *et al*. [18] found ten cases where genetic variants associated with both methylation and expression, but they only examined gene expression data for fewer than 100 genes in these comparisons in a subset of the sample, while Gibbs *et al*. [5] found that approximately 5% of SNPs in their study were significant as both meQTLs and eQTLs. Also, Gibbs *et al*. [5] find proportionally similar number of QTLs for methylation and gene expression, while we find more eQTLs. A potential explanation for the greater overlap obtained in our data is that our study examines one cell type in comparison to heterogeneous cell-types in human brain tissue samples used in both other studies [5,18].

Characterizing the genetic control of methylation and its association to the regulation of gene expression is an important area for research, critical to our understanding of how complex living systems are regulated. Our study has the potential to help disease mapping studies, by informing the phenotypic consequences of this variation. Altogether, of the 173 genes with proximal meQTLs in our study, eighteen genes were previously reported to be differentially methylated in cancer, in other diseases, or across multiple tissues (see Table S4 in Additional file 1). Furthermore, thirty of the meQTL associations reported in our study were also observed in human brain samples [5]. These findings provide a framework to help the interpretation of GWAS findings and improve our understanding of the underlying biology in multiple complex phenotypes.

## Conclusions

Our results, together with recent findings of heritable allele-specific chromatin modification [25,47] and transcription factor binding [26,49] demonstrate a strong genetic component to inter-individual variation in epigenetic and chromatin signature, with likely downstream transcriptional and phenotypic consequences. Importantly, we found an enrichment for SNPs that affect both methylation and gene expression, implying a single causal mechanism by which one SNP may affect both processes, although such shared QTLs represent a minority of both meQTLs and eQTLs. Our data also have implications for the functional interpretation of mechanisms underlying association of genetic variants with disease.

## Materials and methods

### Methylation data

DNA was extracted from lymphoblastoid cell lines from 77 individuals from the Yoruba (YRI) population from the International HapMap project (60 HapMap Phase 1/2 and 17 HapMap Phase 3 individuals). Lymphoblastoid cell lines were previously established by Epstein-Barr Virus transformation of peripheral blood mononuclear cells using phytohemagluttinin. We obtained the transformed cell lines from the Coriell Cell Repositories. Methylation data were obtained using the Illumina HumanMethylation27 DNA Analysis BeadChip assay. Methylation estimates were assayed using two technical replicates per individual and methylation levels were quantile normalized across replicates [28]. At each CpG-site the methylation level is presented as *β*, which is the fraction of signal obtained from the methylated beads over the sum of methylated and unmethylated bead signals. We considered different approaches to normalizing values across replicates, as well as using the log of the ratio of methylated to unmethylated signal instead of *β*, and found the results robust to normalization procedure, measure of methylation, and across technical replicates (see Additional file 1). The methylation data are publicly available [50] and have been submitted to the NCBI Gene Expression Omnibus [51] under accession no. [GSE26133].

We mapped the 27,578 Illumina probes to the human genome sequence (hg18) using BLAT [52] and MAQ [53]. We selected 26,690 probes that unambiguously mapped to single locations in the human genome at a sequence identity of 100%, discarding probes that mapped to multiple locations with up to two mismatches. We excluded a further 4,400 probes that contained sequence variants, including 3,960 probes with SNPs (from the 1,000 genomes project [54], July 2009 release, YRI population) and 440 probes which overlapped copy number variants [55]. This resulted in a final set of 22,290 probes (21,289 autosomal probes) that were used in all further analyses. The 22,290 probes were nearest to the TSSs of 13,236 Ensembl genes, of which 12,901 genes had at least one methylation CpG-site within 2 kb of the TSS.

Bisulfite sequencing was performed in the *C21orf56 *region for eight individuals. DNA was bisulfite-converted using the EZ DNA Methylation-Gold Kit (Zymo Research). PCR amplification was performed using primers designed around CpG-site cg07747299 from the HumanMethylation27 array and the nearest CpG island in the region (using Methyl Primer Express from Applied Biosystems) for a total of 411 bp amplified in the 5' UTR of the *C21orf56 *gene. PCR products were sequenced and cytosine peak heights compared to overall peak height were called using 4Peaks Software.

### Gene expression data

RNA-sequencing data were obtained for LCLs from 69 individuals in our study from [24]. The methylation and RNA-sequencing data were obtained from the same cultures of the LCLs. RNA-sequencing gene expression values are presented as the number of GC-corrected reads mapping to a gene in an individual, divided by the length of the gene. In the methylation to gene expression comparisons we split genes into quantiles based on the mean gene expression per gene. For the eQTL analyses, RNA-sequencing data were corrected and normalized exactly as in [24]. Of the 22,683 genes in the original study, 10,167 autosomal genes had both gene expression counts and methylation CpG-sites within 2 kb of the TSS.

### Genotype data

HapMap release 27 genotype data were obtained for 3.8 million autosomal SNPs in HapMap (combined Phase 1/2 and 3). Missing genotypes were imputed by BIMBAM [56] using the posterior mean genotype. Non-polymorphic SNPs were excluded, reducing the set to 3,035,566 autosomal SNPs for association analyses.

### Statistical analysis

Spearman rank correlations were used to assess co-methylation between probes and to compare methylation and gene expression. We used 10,000 permutations of the gene expression to methylation assignments to assess the enrichment of negatively and positively correlated genes in the 25% and 5% tails within genes. Wilcoxon rank-sum tests were used to compare probe means and variances for subsets of probes.

### Association analyses

Genome-wide association was performed using the methylation values at each CpG-site as phenotypes and three million autosomal SNP genotypes. We used least squares linear regression with a single-locus additive effects model, where we estimated the effect of the minor SNP allele on the increase in methylation levels. Prior to the association analyses, we normalized the methylation values at each CpG-site to N(0, 1) and applied a correction using principal component analysis regressing the first three principal components to account for unmeasured confounders following similar approaches to reduce expression heterogeneity in gene expression experiments [24,42,43] (see Additional file 1). Sex-specific analyses were performed using sex as a covariate and assessing the significance of the sex by additive-QTL interaction term.

We assessed the enrichment of association at SNPs and probes that were previously reported to be associated with methylation [7,8,15-18] and at SNPs within 200 kb of genes known to affect DNA methylation (Table S3 in Additional file 1). We also compared genetic variation to normalized variation in the principal components loadings for the autosomal methylation data (see Additional file 1). Results from the 180 *cis *meQTLs are available online [50].

### FDR calculation

We performed genome-wide permutations to assess the significance of the genome-wide association results in the least-square linear regressions. We permuted the methylation values for the 21,289 autosomal probes (phenotypes), performed genome-wide association on the 21,289 permuted and normalized phenotypes, and repeated this procedure for 10 (*cis*-analyses) or 1 (*trans*-analyses) replicates selecting the best signal per probe per replicate. Results are presented at an FDR of 10%, meaning that an estimated 10% of the meQTLs are false positives. Results for additional FDR thresholds are shown in Additional file 1. FDR was calculated as the fraction of significant hits in the permuted versus the observed data at a given *P-*value threshold. The association analyses and FDR calculations were performed for all autosomal principal components and CpG-sites in the methylation data, and for all autosomal genes in the RNA-sequencing data.

### Hierarchical model

We fitted a Bayesian hierarchical model [22] to test whether meQTLs were over-represented in transcription factor binding sites, histone-modifications, and with respect to distance to the probe. We extended the model to fit the methylation data, where the reference point was the location of the methylation probe. Each annotation category that we examined was included in the model while accounting for distance effects.

### Genome annotations

Genome annotation data were obtained from UCSC (hg18). Histone modification data were obtained from ChIP-seq reads from the ENCODE project (Bernstein lab) for GM12878 for seven histone marks. Histone modification categories were based on estimated peaks in the read-depth distribution (see Additional file 1).

Transcription factor binding site locations were estimated using the algorithm CENTIPEDE [40,57]. For the results presented here, CENTIPEDE started by identifying all matches in the genome to a large number of transcription factor binding motifs obtained from the TRANSFAC and JASPAR databases. It then estimated which potential binding sites are actually occupied by transcription factors in LCLs, by incorporating input data from sequence conservation, location with respect to nearby genes, and cell-specific experimental data, including DNaseI data. We used 1,136,620 non-overlapping sites from 751 transcription factor motif matches that overlapped 1,913 CpG-sites.

## Abbreviations

CEPH: Centre d'Etude du Polymorphisme Humain; CGI: CpG island; ChIP-seq: chromatin immunoprecipitation followed by sequencing; CpG: cytosine-phosphate-guanine; DIP2B: disco-interacting protein 2 homolog B gene; DNMT: DNA methyltransferase; eQTL: expression quantitative trait locus; FDR: false discovery rate; LCL: lymphoblastoid cell line; meQTL: methylation quantitative trait locus; QTL: quantitative trait locus; SNP: single nucleotide polymorphism; TSS: transcription start site; UCSC: University of California Santa Cruz genome browser; YRI: Yoruba.

## Competing interests

The authors declare that they have no competing interests.

## Authors' contributions

JTB, JKPr, and YG wrote the paper and interpreted the results. JKPr and YG designed the study. JTB analyzed the data. AAP performed bisulfite sequencing and sample preparation. JKPi mapped and processed the RNA-sequencing data, and helped with the analyses. DJG mapped and processed the histone modification data. RP-R and JFD provided estimates for the transcription factor binding sites. All authors read and approved the final manuscript.

## Supplementary Material

Additional file 1 **Supplementary material**. Contains Supplementary Methods and Results, Supplementary Figures 1-11, and Supplementary Tables 1-4. Click here for file
