# Supplementary material for: DNA methylation patterns associate with genetic and gene expression variation in HapMap cell lines
Source: Genome Biol. 2011 Jan 20;12(1):R10. doi: 10.1186/gb-2011-12-1-r10 (PMC3091299; doi:10.1186/gb-2011-12-1-r10)
Supplement: Additional file 1 — Supplementary material. Contains Supplementary Methods and Results, Supplementary Figures 1-11, and Supplementary Tables 1-4. [file gb-2011-12-1-r10-S1.PDF]

# Supplementary Material: DNA methylation associates with genetic and gene-expression variation in HapMap samples

Jordana T. Bell, Athma A. Pai, Joseph K. Pickrell, Daniel J. Gaffney, Roger Pique-Regi, Jacob Degner, Yoav Gilad, Jonathan K. Pritchard

January 18, 2011

## Contents

**1 Methylation Data** **2**
1.1 Methylation Measures . . . 2
1.2 Replicates and normalization . . . 2
1.3 Confounders . . . 3

**2 Association analyses and results** **3**
2.1 Unobserved confounders . . . 3
2.2 Sex-specific analyses . . . 4
2.3 Genome-wide tests of association . . . 4

**3 Genome Annotations** **6**
3.1 Genes and CpG islands . . . 6
3.2 Histone Modifications and Motifs . . . 6

**4 Hierarchical Model** **7**

**5 Supplementary Figures** **8**

**6 Supplementary Tables** **19**

# 1 Methylation Data

## 1.1 Methylation Measures

We examined the intensity signal obtained from the methylated (M) and unmethylated (U) beads at each CpG-site in the assay. We compared the standard error in the signal from both M and U beads per individual and found no prominent outliers across individuals. We observed slightly higher but significant methylation means from probes that fell in the red (9,178 probes) compared to the green (11,969 probes) channel (0.259 vs 0.233, Wilcoxon rank sum test P-value $< 1e\text{-}7$), indicating a potential bias in the assay that has not been reported so far [29]. Finally, we considered different measures to summarize methylation values. We used two measures consistently throughout the study - $\beta$ (suggested by Illumina) and log(ratio):

$$\beta = \frac{M}{M+U+100}$$

$$log(ratio) = log(\frac{M}{U})$$

In general, the results obtained using $\beta$ were very similar and in some cases identical to the results with log(ratio). For example, we obtain similar and slightly more significant negative correlations between gene-expression and methylation when we use the log(ratio). We present results using $\beta$ throughout the study.

## 1.2 Replicates and normalization

The methylation assay was performed twice using 2 replicates per individual, where individuals were assigned to methylation chips in random order across the 2 replicate sets. Within-sample replicate correlation (Pearson rho = 0.98) exceeded the correlation observed between two unrelated samples (Pearson rho = 0.91).

Within replicate set, we obtained the individual autosomal methylation means and the first 3 principal components (PCs) to test for differences in the distribution of the 77 genome-wide methylation profiles according to methylation chip, order of the sample on the chip, HapMap version, and sex. We found a significant difference in the distribution of the first 2 principal component scores across methylation chip.

To combine data across the two replicates per individual, we quantile normalized the data across the 2 replicates. We also tried other approaches to combine and normalize the data across the 2 replicates and obtained similar results to those presented in the main text. Briefly, we averaged across the two replicates, we quantile normalized the replicate 2 data to the empirical distribution in replicate 1, and we fit a linear model controlling for methylation chip and individual and obtained the fitted values corresponding to the data in replicate 1. In all cases the gene-expression to methylation mean rank-correlation within individuals was -0.4 and similar estimates were obtained when examining data from

the individual methylation replicates alone. Of the 180 cis meQTLs presented in the main text, 165 overlapped with the FDR10% cis results from the other combined and normalized datasets, and 120 overlapped with the FDR10% cis results in replicate 1 alone.

### 1.3 Confounders

To test for potential confounders in the data we compared overall methylation levels per individual (quantile-normalized across 2 replicates) and applied principal component analyses (PCA) to the autosomal and X-chromosome methylation data. We examined the distribution of the first 5 PCs across potential covariates in our sample. For the X-chromosome, the first principal component, which explained over 50% of the variation, was clearly gender and corresponded to higher methylation levels in females versus males. For the autosomal data, we did not observe differences with gender, but we did see significant differences for Hapmap phase (2 vs 3) in the distribution of the first autosomal principal component scores. The first principal component explained 22% of the variation in methylation and was correlated with overall mean methylation levels (rank correlation = 0.64). We then repeated the procedure comparing the autosomal methylation data with data from Choy et al (2008) [30] in 60 (HapMap I+II) of the 77 individuals in our sample. In the latter case, we examined correlations between the first 5 PCs and 30 variables from Choy et al.: EBV, EBV2, EBV-Expansion, mtDNA, growth, ATP levels, and 14 variables in the supplementary file labelled: isotype controlled cell-surface markers. We found no significant ($p < 0.001$) correlations for any of those factors agains the first 5 PCs and the individual methylation means.

## 2 Association analyses and results

### 2.1 Unobserved confounders

To account for potential unknown confounders in the association analyses, we regressed out autosomal principal components from the methylation data. This procedure was applied because similar methods have been proposed to reduce the effects of confounding factors in gene expression experiments to increase power to detect expression quantitative loci [42, 24]. We removed PCs by calculating the eigenvectors of the $77 \times 77$ individual correlation matrix in R, and regressing out the eigenvector loadings by linear regression. Although the PCs that we regress out may represent biological quantities of interest, they may also capture technical assay variability which we wish to minimize and increase the power to detect variants associated with methylation. Therefore, in the association analyses, we adopted the following procedure: we quantile normalized the methylation values for each autosomal methylation probe to N(0,1), then regressed the first 0 - 10 PCs from these data, and quantile normalized the residuals from the regression to N(0,1). We present results for the methylation residuals from the first three PCs, which explain 33% of the variation and

maximize the number of meQTLs in our data.

## 2.2 Sex-specific analyses

Sex-specific QTL analyses were performed by fitting the following model:

$$y_i = \mu + \beta_0(gender) + \beta_1(SNP_{additive}) + \beta_2(gender * SNP_{additive}) + \epsilon_i$$

where $y_i$ is the methylation value at a CpG-site in individual $i$, $\mu$ is the mean methylation in males, and $\beta_0$ is the coefficient for the sex effect, $\beta_1$ is the additive effect of the SNP on methylation, and the interaction term $\beta_2$ takes into account the possibility that the effect of the QTL may have a different direction in males and females. We assessed the significance of $\beta_2$ by permuting the gender labels, while keeping the genotype and methylation data constant for one genome-wide permutation.

## 2.3 Genome-wide tests of association

We first compared genetic variants to variation in the principal component loadings from the autosomal methylation data. Principal components loadings were normalized to N(0,1) prior to association analyses. The most significant findings was obtained for SNP rs10876043, which had a genome-wide significant association with variation in the first principal component of methylation (P = 4.51 $\times 10^{-9}$), and which also showed a modest association with average genome-wide methylation level (P = 4.00 $\times 10^{-5}$). The first principal component explained 22% of the variation in methylation and was correlated with overall mean methylation levels (rank correlation = 0.64). This SNP lies within the intron of the gene *DIP2B*, which contains a DMAP1-binding domain, and has been previously proposed to play a role in DNA methylation [41]. The peak results at a false discovery rate of 10% (P = $10^{-7}$) from the association analyses comparing genetic variation to normalized variation in the overall mean and principal components are shown in Supplementary Table S1.

We next assessed genome-wide associations between SNP genotypes and methylation levels at individuals CpG-sites. At a false discovery rate (FDR) of 10% (corresponding to a P-value of 2.05e-10) 37 CpG-sites showed evidence for association with methylation status, and 30 of those were on the same chromosome as the CpG-site (Supplementary Table S2). At an FDR of 5% there were 35 CpG-sites with evidence for association. For 27 of the 30 CpG-sites, we observed that the majority of signal was obtained from SNPs within 50kb of the probe. The 37 CpG-sites form 44 CpG-SNPregion association, where we defined each SNP region using bins of 1Mb (there were no adjacent bins). Results are presented from the F-statistics testing the fit of single-locus additive model to the null model with no genetic effects. The analyses were then repeated using Bayesian linear regression [58] implemented in BIMBAM [56]. We obtained the corresponding Bayesian linear regression results at a posterior probability threshold of -$\log_{10}$ (Bayes factor) of 6, which were highly

correlated with the P-values from the linear regressions (data not shown).

We examined the *trans* results in detail. Overall, the evidence for association in *trans* was weak, with significant signals obtained for probes near to the following 10 genes: *UBL5, UGDH, C16orf52, MACF1, ATG10, CYP4X1, HOXB13, HSD17B12, RANBP6, and FAM54A* (Supplementary Table S2). We also searched to identify SNPs which were enriched for *trans* association signals at more relaxed significance thresholds. We selected a list of SNPs that fell within 200kb of the TSS/TES of 14 candidate genes, as well as previously identified SNPs with significant or suggestive evidence for association to methylation (Supplementary Table S3). At each SNP of interest we compared the number of genome-wide associations obtained at a nominal P < 1e-6 with the goal of identifying loci that may affect methylation genome-wide. First, we found that rs8075575, which was 150kb from *ZBTB4*, was enriched for association with cg24181591 (*EIF5A/ENSG00000132507*) on the same chromosome. Second, we also found suggestive signals at rs8092996 165kb from *MBD2* with probe cg06781209 (*FADS2*) on chromosome 11, hyper-methylation at which has previously been linked with hyperhomocysteinemia in mice [59]. Three previously reported [5] significant distal associations were also observed for SNPs rs7225527 (38kb from gene *RHBDL3*) and methylation at probe cg17704839 in gene *UBL5* that encodes ubiquitin-like protein, and for SNPs rs2638971 (106kb from gene *DDX11*) and rs17804971 (49kb from gene *DDX12*) and methylation at probe cg18906795 in gene *RANBP6*, which may function in nuclear protein import as a nuclear transport receptor. Finally we also had association signals at rs17123980 192kb *DNMT3B* with probe cg10968815 (*BPIL1*) on chromosome 20, and at rs12984113 and rs10424964 - both around 22kb from *DNMT1* - with probe cg06177968 (*WDR55*) on chromosome 5.

When we examined the association signal at previously identified SNPs, we found 3 SNPs (rs3881953, rs12743401, and rs12734338 on chromosome 1), which were associated with cg09067967 (*UGDH*) on chromosome 4. Interestingly, Boks et al [16] find these same 3 SNPs to be moderately associated with TDGF1_P428_R on chromosome 3, from the Illumina GoldenGate Methylation assay (for which we have no data). Given the discrepancy of the cg09067967 results, it is doubtful that these findings represent associations of biological interest. However, BLAT results for probe sequence cg09067967 give a single perfect match on chromosome 4, which suggest that cross-hybridization to multiple locations is unlikely to contribute to these discrepant association results. Finally, we examined SNPs for an enrichment of association signal across multiple probes at $P < 10^{-2}$ and found that the majority of SNPs influenced two probes, with a maximum of 13 probes for rs12565445 and rs625372 (near *SIGLEC1*).

We next focused on associations that only included SNPs in the vicinity of the CpG-sites, or *cis* meQTLs. Overall, we observed 180 *cis* meQTLs at FDR of 10%, and 131 and 75 meQTLs at FDR of 5% and 1%, respectively. The list of results that surpassed the FDR10% threshold for these analyses is presented online (http://eqtl.uchicago.edu/Met.results). For these *cis* least squares linear regressions we present the -$\log_{10}$(P-value) obtained from the single-locus additive effect coefficients.

We searched for previous evidence relating the 173 genes with *cis* associations, to methylation or imprinting. We found 2 genes, with previous evidence for imprinting (listed in the Imprinted Gene Catalogue, see section below) - *MEST and CPA4*. Another two genes may also exhibit imprinting effects: *H6PD* gene-expression is up-regulated in bovine parthenotes (cells originating from parthenogenesis) suggesting imprinting effects [60], and in the mouse *Slc28A2* is located near to the imprinted gene Gatm, but has not itself been found to be imprinted in the mouse placenta [61]. Of the 173 genes, 18 genes had previous evidence for differential methylation in cancer cells, or across multiple tissues, or following tissue differentiation, or in disease (Supplementary Table S4). The differentially methylated region reported for *PERP* (Supplementary Table S4) may in fact be due to a mRNA-like piRNA between the *PERP* and *KIAA1244*, which has been found in the mouse and shows evidence for transcriptional regulation by methylation [62]. Finally, *BAG1* is another gene potentially linked to methylation - it is an oncogene, where hypo-methylation decreases gene-expression [63], and recent evidence suggests that *DNMT1* and *DNMT3B* activate *BAG1* expression via recruitment of CTCF ligand and modulation of promoter histone methylation [64].

# 3 Genome Annotations

## 3.1 Genes and CpG islands

Gene annotations and DNA sequence features information were obtained from the UCSC genome browser. CpG Islands track information [33] was obtained from UCSC. Each methylation probe was assigned to a gene using gene annotation data from Ensembl. We assigned each probe to the nearest gene and for most analyses restricted the probe set to those that were within 2kb of the TSS for the nearest transcript.

Data on imprinted genes were obtained from the Imprinted Gene Catalogue (IGC) at http://igc.otago.ac.nz/ for a total of 61 genes with evidence for imprinting in humans [65]. We cross-referenced the gene name from IGC to gene annotation data from Refseq. Refseq gene information was downloaded from UCSC, hg18. We then selected only genes for which there was a CpG-site within 2.5 kb of the TSS, for a total of 153 CpG-sites in 33 autosomal imprinted genes.

## 3.2 Histone Modifications and Motifs

Histone modification ChIP-seq data were obtained from the Encode project from the CEPH HapMap LCL (GM12878) in the UCSC genome browser. We obtained data for genome-wide distribution of reads from ChIP-seq histone modifications for 7 modifications: H3K9ac, H3K27ac, H3K27me3, H3K4me1, H3K4me2, H3K4me3, H4K20me1. Each histone genome-wide distribution of reads was smoothed using a bandwidth of 100 and peaks were assigned using a stringent threshold of 1.0.

Transcription factor binding site locations were estimated using the algorithm CENTIPEDE (RPR, JFD, et al, in prep.). For the results presented here, CENTIPEDE started by identifying all matches in the genome to a large number of transcription factor binding motifs obtained from the TRANSFAC and JASPAR databases. It then combined various types of information to estimate which potential binding sites are actually occupied by transcription factors in LCLs. The input data included sequence conservation, location with respect to nearby genes, and cell-specific experimental data–most importantly DNase-seq data from ENCODE (Crawford lab) and our own lab. Comparisons to ENCODE ChIP-seq data for six transcription factors show that CENTIPEDE achieves high specificity. We used 1,136,620 non-overlapping sites genome-wide from 751 transcription factor motif matches (with some redundancy) that overlapped 1,913 CpG-sites. For CTCF, there were 16,395 non-overlapping sites genome-wide.

# 4 Hierarchical Model

We applied the hierarchical model of Veyrieras et al. [22] to the *cis* meQTL data, to estimate the effects of different annotation categories on the probability that a SNP is a meQTL. We tested whether the following categories were enriched for meQTLs: eQTLs, histone modification peaks, transcription factor binding motifs and CTCF binding motifs in particular, and proximity to the methylation probe. We estimate the effect of each annotation class, comparing SNPs that fall in the annotation category relative to the abundance of meQTLs in the null annotation category (or the furthest 5' distance bin from the probe under a distance-only model).

We fit the HM with respect to the methylation probe location, rather than the TSS as in the original study. We examined the region from -50,000 to +50,000 around the probe, using distance bins of sizes 10,000bp, and then 1,000 bp for distances within 10,000bp around the probe. For each annotation category that we tested, we also included the distance model to control for the effects of distance. Results are presented as the maximum-likelihood estimated of log odds ratio and corresponding 95% confidence intervals.

# 5 Supplementary Figures

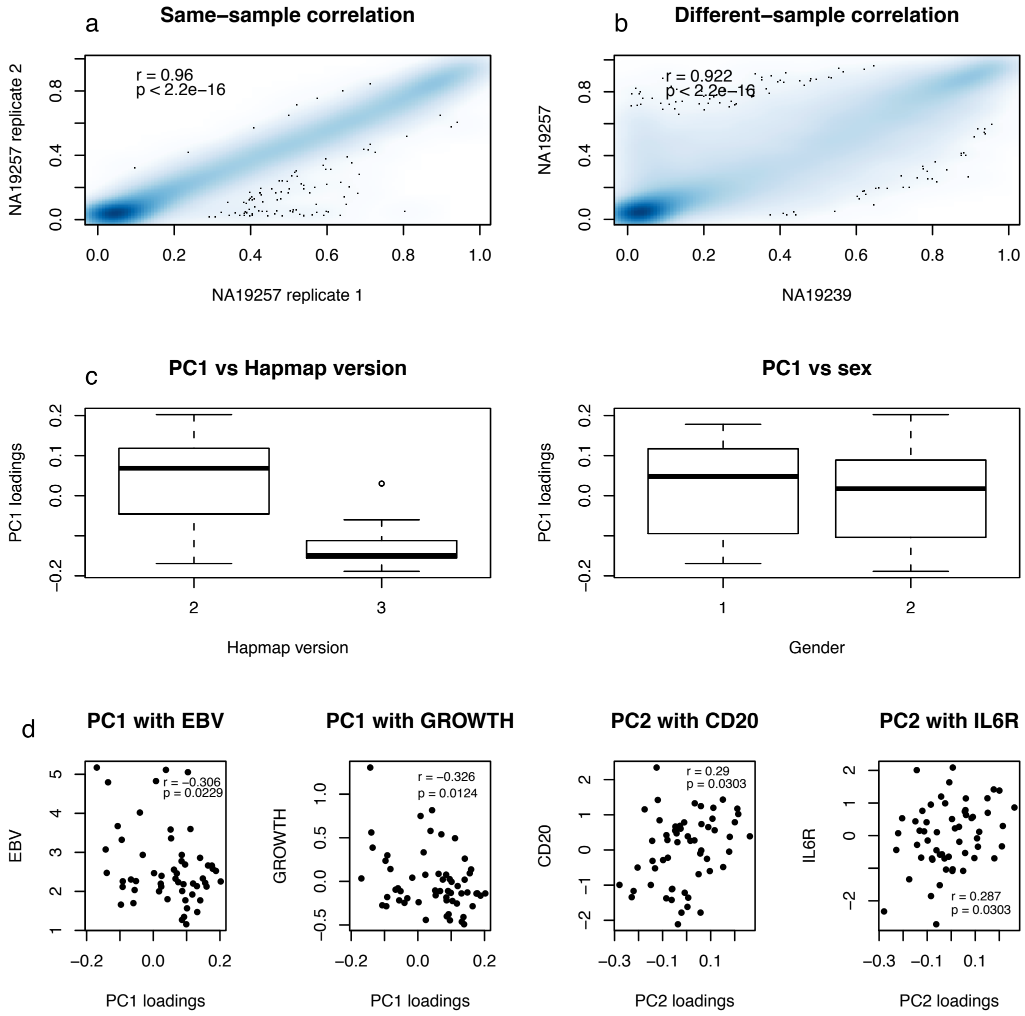


Figure S1. Autosomal methylation data and testing for confounders. Principal components were obtained from the individual-correlation matrix, where we obtained all pair-wise individual correlations. (a) Correlations between two replicates of the same sample were always higher than (b) correlations between two randomly selected samples. (c) Distribution of the first methylation PC across potential covariates. (d) The most significant results comparing the distribution of the first five methylation PCs across variables tested from Choy et al (2008).

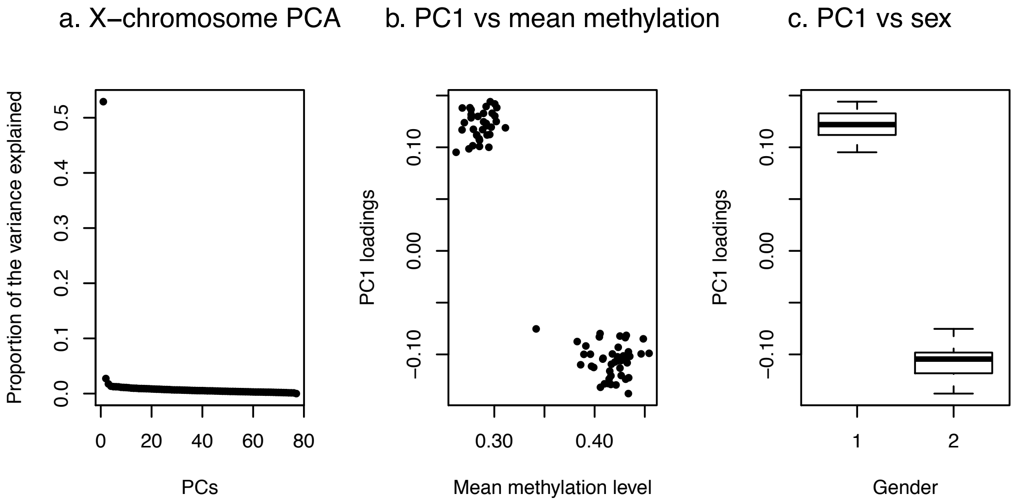


Figure S2. Principal component analysis of the X-chromosome methylation data. (a) The proportion of the variance explained by the principal components. (b) The first PC corresponds to mean overall methylation per individual. (c) Gender against the first PC loadings, showing that females (2) have higher mean methylation than males on the X chromosome.

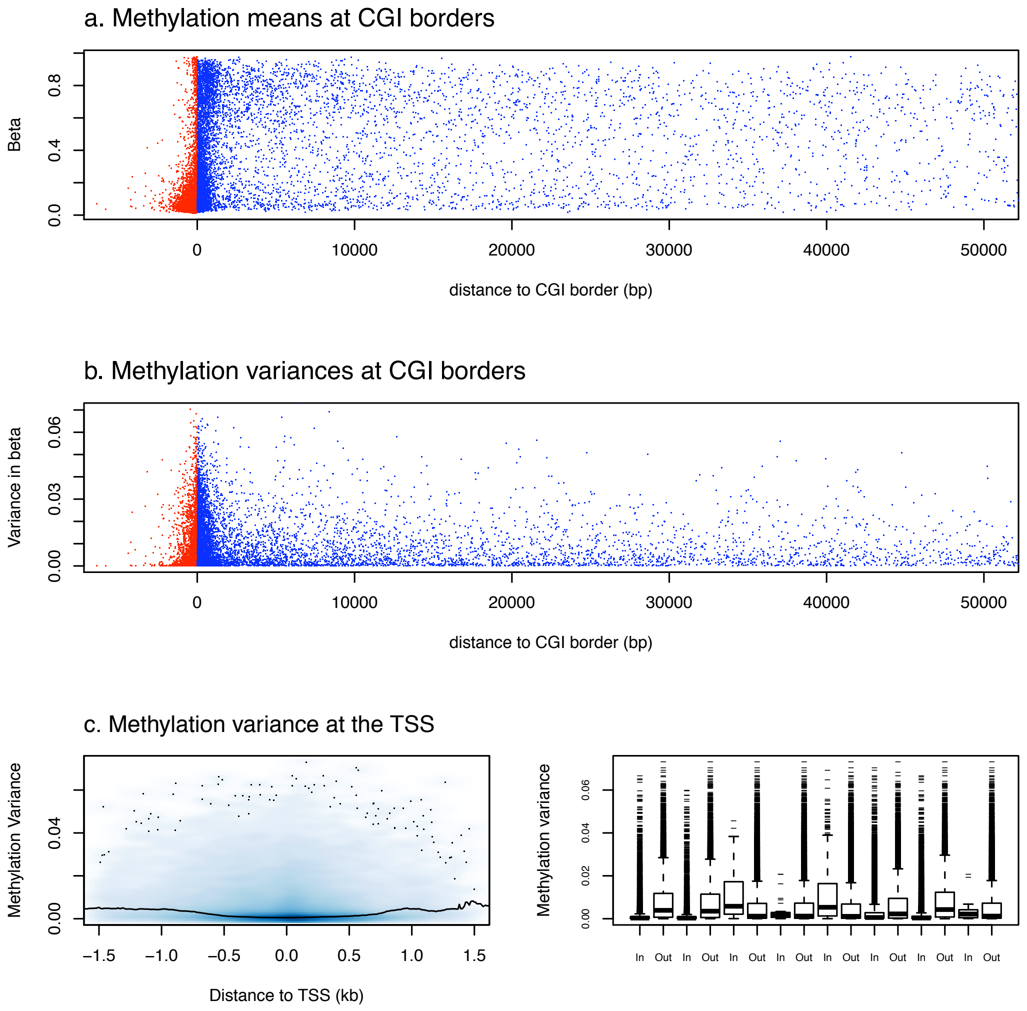


Figure S3. Methylation variances and methylation with respect to CpG Islands. Methylation (a) levels and (b) variances are shown according to position of the CpG-site relative to CGI for all probes within 50kb of the CGI border (90% of probes). Probes inside of CGI are in red and probes outside of CGIs are in blue. Although we observed marked methylation differences for probes in or out of CGIs, we did not detect a significant relationship between methylation and CGI border (including CpG island shores [34]). Higher-resolution methylation data may be needed to detect CpG island shore effects. Methylation variances are shown with respect to (c) distance to the TSS and (d) histone modifications.

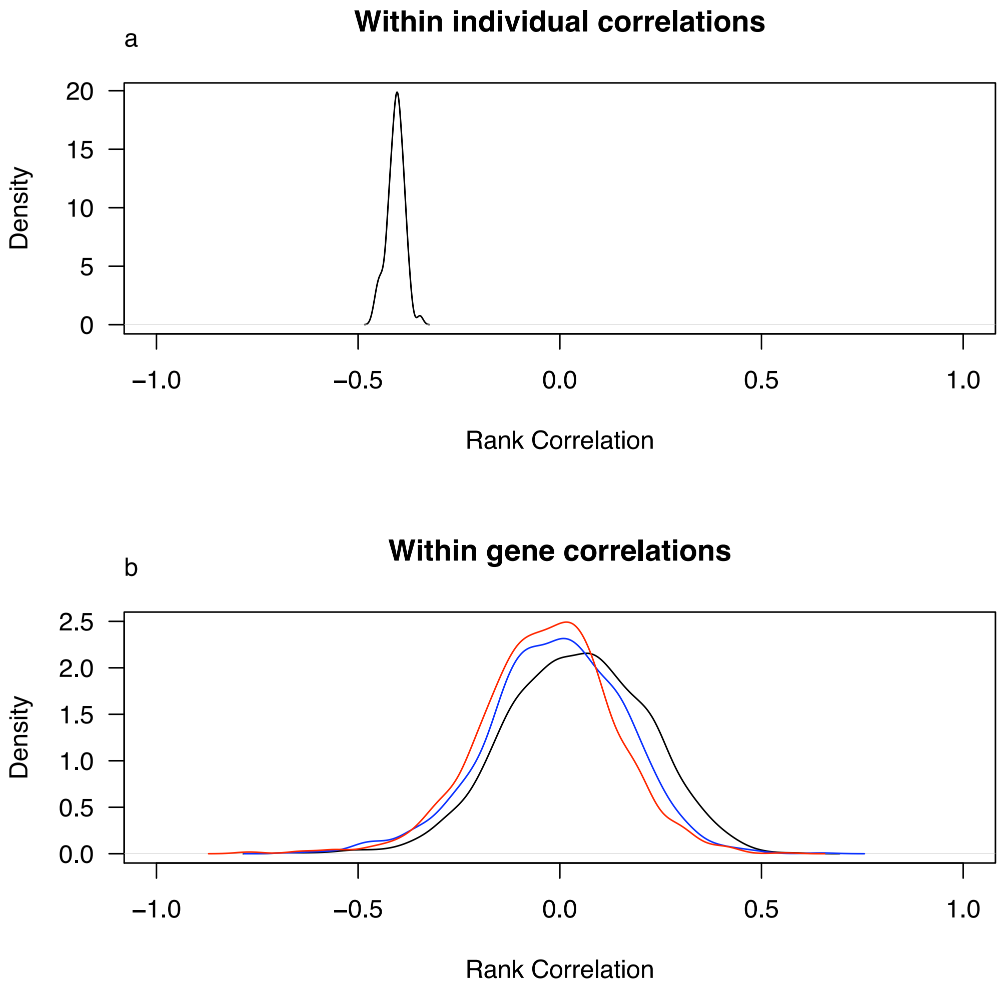


Figure S4. Distribution of correlation coefficients comparing methylation to gene-expression. (a) Mean correlation of -0.407 within 77 individuals, across 12,670 genes. (b) Distribution of correlation coefficients in 10,532 expressed (in $>$ 50% sample) genes, across 77 individuals (black). We used 10,000 permutations of the gene-expression to methylation assignments to assess the enrichment of negatively and positively correlated genes in the 25% and 5% tails. A significantly higher proportion of negatively correlated genes was seen in the observed data and the enrichment was greater for negative compared to positive correlations. We then restricted the methylation probes to subsets of variable probes (the top 50% of variable probes) and were within 1.2kb of the TSS (between 400bp away and 800bp inside the gene, from Fig. 1b). Within this probe subset there was a higher proportion of negatively-correlated probes that fell either in CGIs (red) or outside of CGIs (blue).

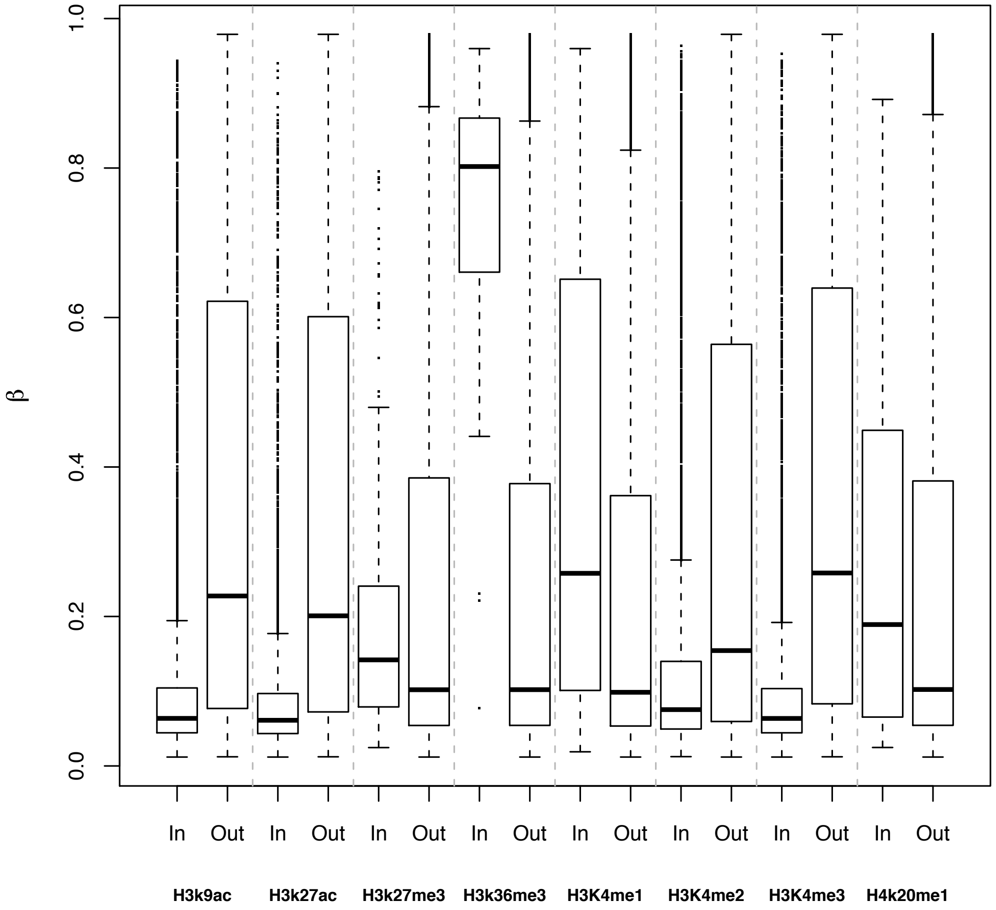


Figure S5. Methylation levels in histone-modification peaks only within 5kb of TSS from the EncodeLCL ChIP-seq data. For the majority of histone modifications there were significant differences in the mean CpG-site methylation levels according to whether the probes were in or out of histone peaks (Wilcoxon rank test, $P < 2e\text{-}16$). The two exceptions were H4k20me1, $P = 0.3414$, and H3k27me3, $P = 0.0212$). These peaks represent a subset of all peaks presented in Fig 1c.

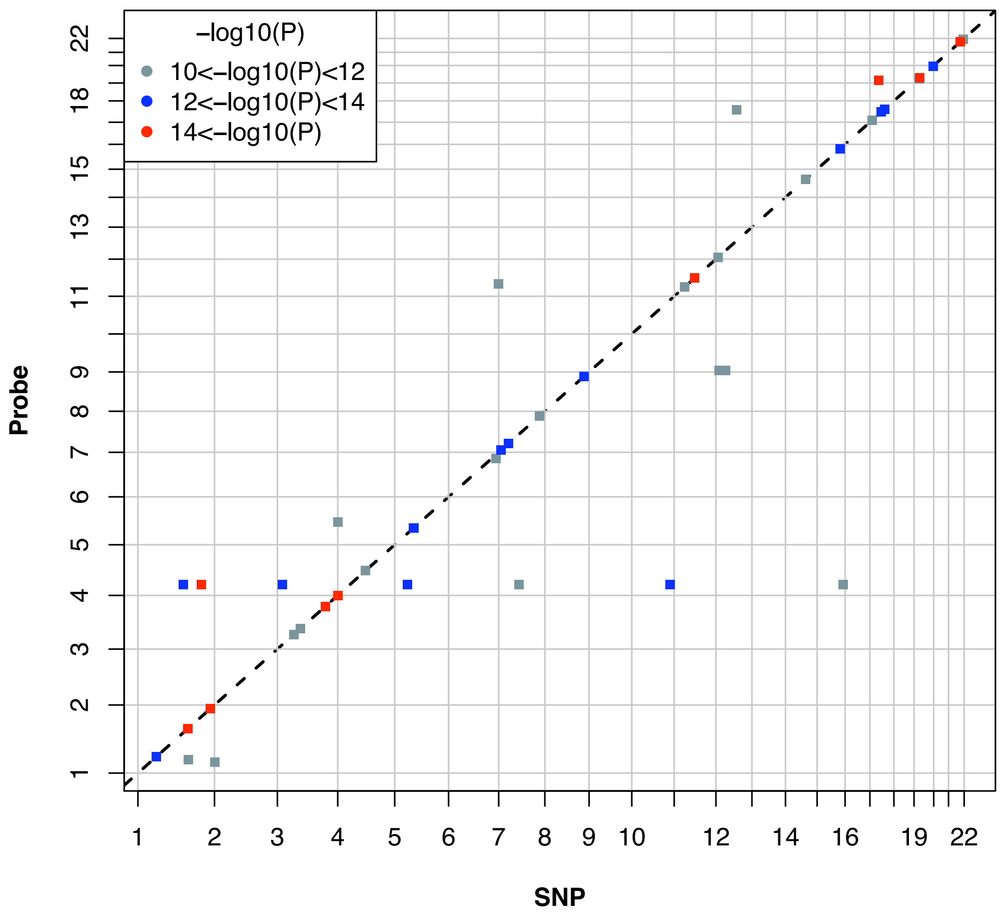


Figure S6. All genome-wide *cis* and *trans* results using the least-squares regressions at an FDR 10% threshold (P-value of 2.05 $\times 10^{-10}$).

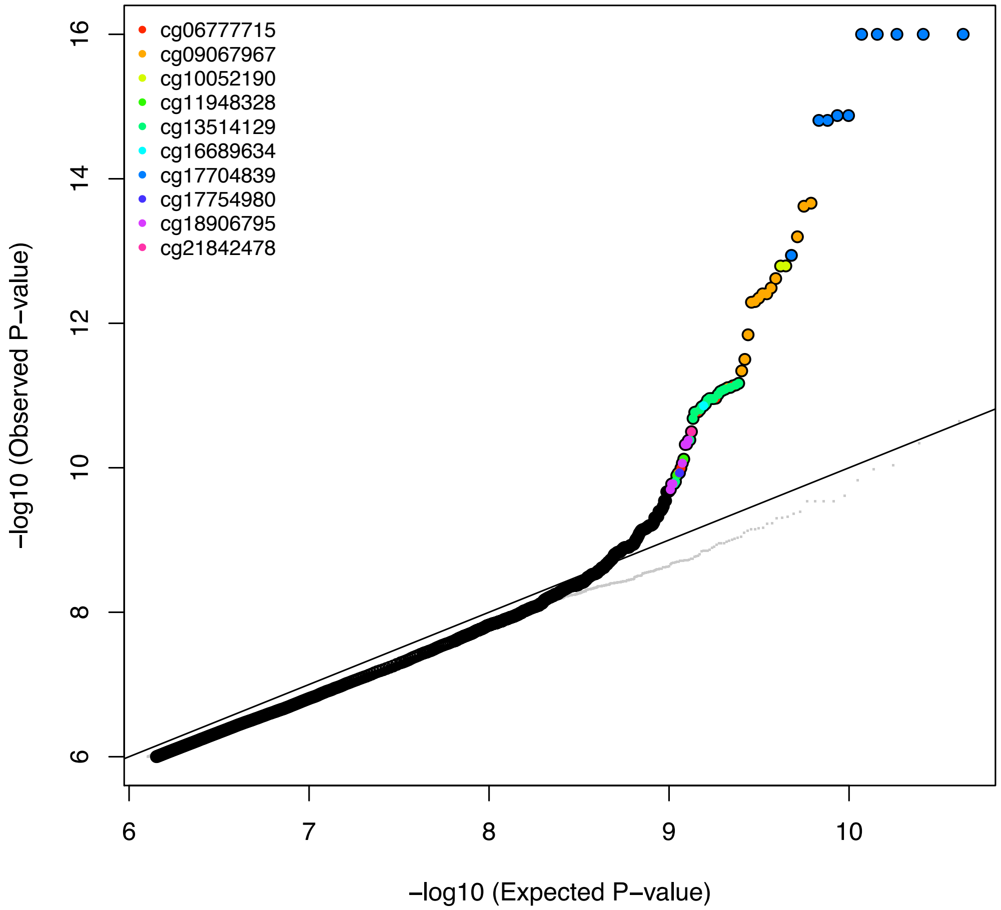


Figure S7. QQ plot of the *trans* methylation association signal. The results plotted here represent all tests on different chromosomes, or on the same chromosome where the SNP was at least 1Mb away from the probe (to reduce potential cis-effects in long range LD regions). Observed results are shown in black (and multi-colored large points) and in grey (small points) are *trans* results from one genome-wide permutation of the data. The tail (above diagonal, $P = 1.2 \times 10^{-9}$) consists of trans association signals in 35 probes, of which the most-associated signals (genome-wide cis and trans FDR of 10%) were observed in 10 probes (see legend). See **Supplementary Table S2** for more details on the trans-associations, including nearest gene information.

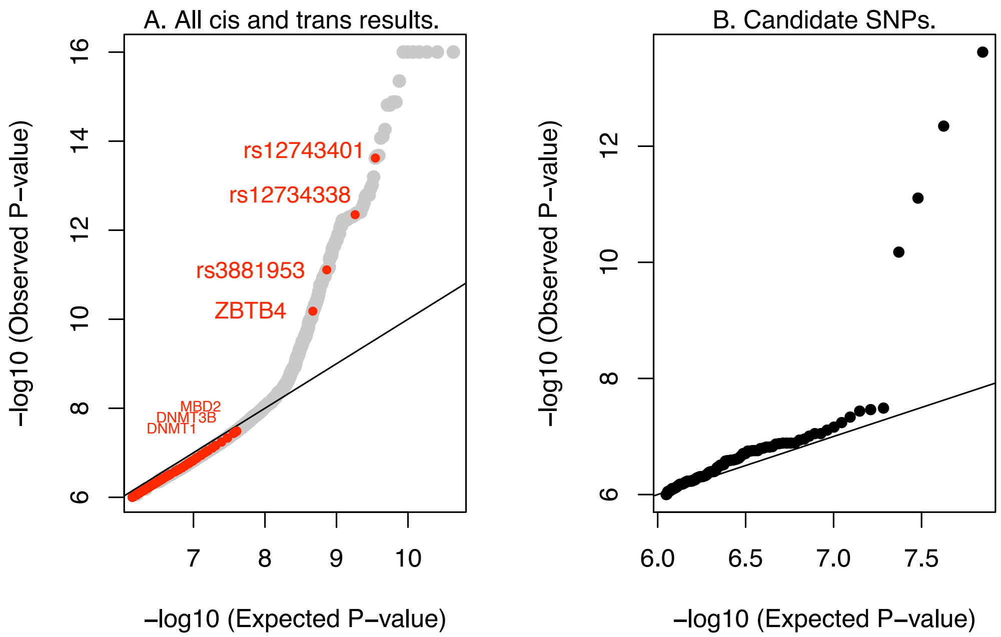


Figure S8. QQ plot of the *cis* and *trans* methylation association signal in candidate SNPs and genes, (a) showing the locations of candidate SNPs relative to all signals, and (b) just focusing on the candidate SNPs. We have highlighted 7 SNPs of interest, of which four fall into candidate genes: rs8075575 (*ZBTB4*) with probe cg24181591 (*EIF5A*) on chromosome 17, rs8092996 (*MBD2*) with probe cg06781209 (FADS2) on chromosome 11, rs17123980 (*DNMT3B*) with probe cg10968815 (*BPIL1*) on chromosome 20, and rs12984113 and rs10424964 (*DNMT1*) with probe cg06177968 (*WDR55*) on chromosome 5. In addition, three SNPs (rs3881953, rs12743401, and rs12734338 on chromosome 1) were associated with cg09067967 (*UGDH*) on chromosome 4, and interestingly, Boks et al found these same three SNPs to be moderately associated with TDGF1_P428_R on chromosome 3. BLAT results for probe sequence cg09067967 give a single perfect match on chromosome 4, which suggests that cross-hybridization to multiple locations is unlikely to have generated the association.

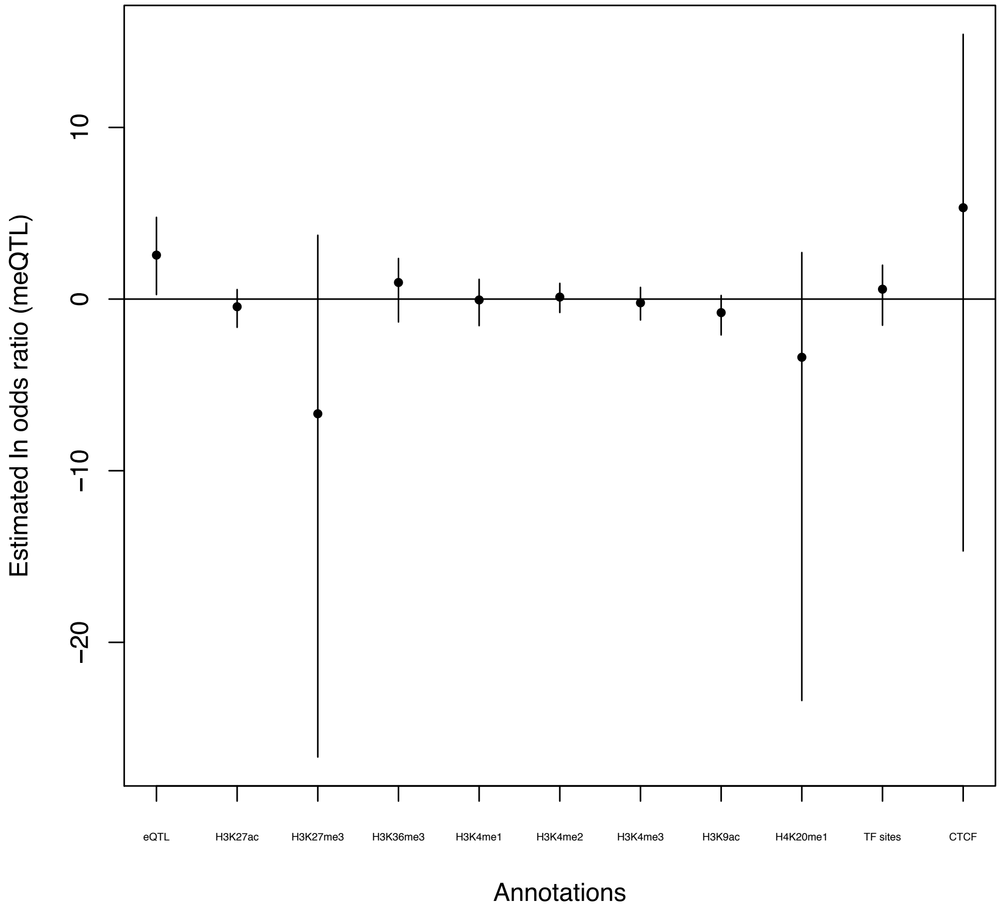


Figure S9. Results from the hierarchical model for annotation categories. Each annotation category was tested while accounting for distance effects. Odds ratio are shown for SNPs that fall into the annotation category relative to SNPs that fall outside that class.

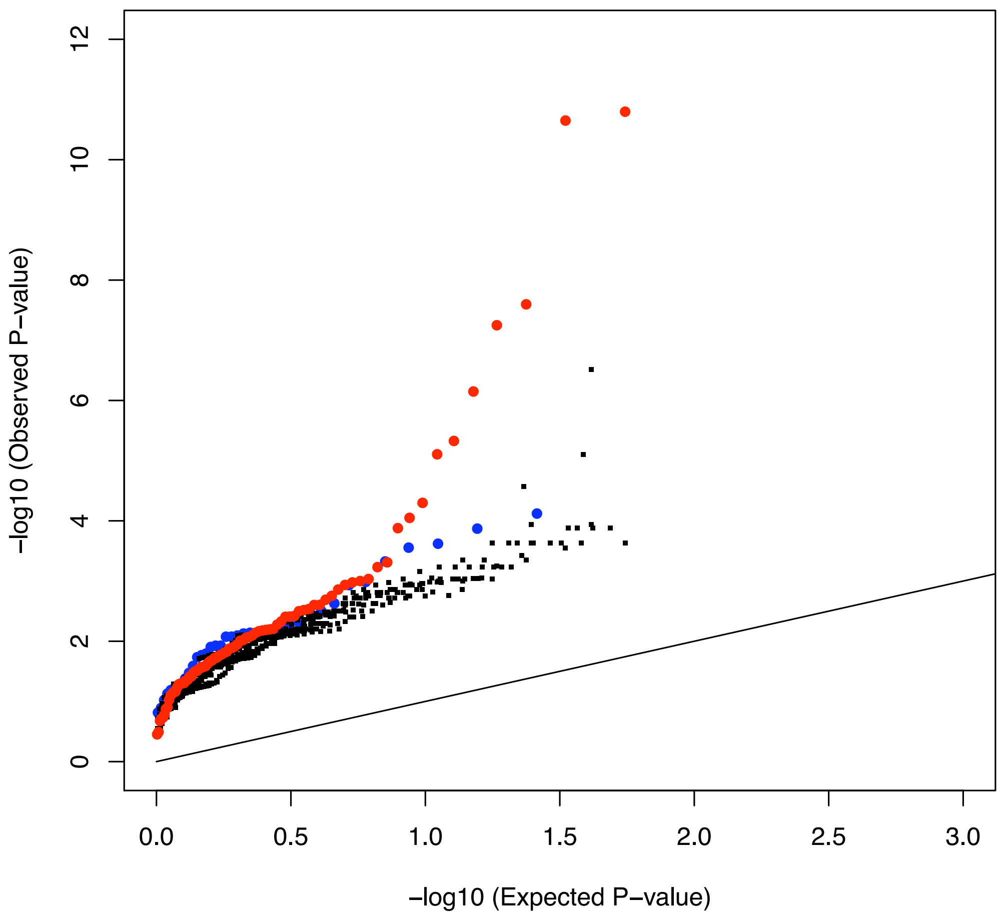


Figure S10. QQplot of meQTL signal in the 45 eQTLs that were only present in the original eQTL results, but disappear in the methylation-residuals. We show the methylation-association P-value for the best SNP per probe, for 82 probes altogether that were assigned to the 45 eQTL genes (red). In blue are the meQTL signals in the 24 eQTLs (38 probes) that were only present in the methylation-residuals, again showing the best methylation-associated SNP per probe. In black are ten permutations of the methylation-regressions, where for each permutation we scored the number of eQTLs that disappeared from the original to methylation-regression eQTLs; we then obtained the best-association-methylation signal in these probes and plot them in black.

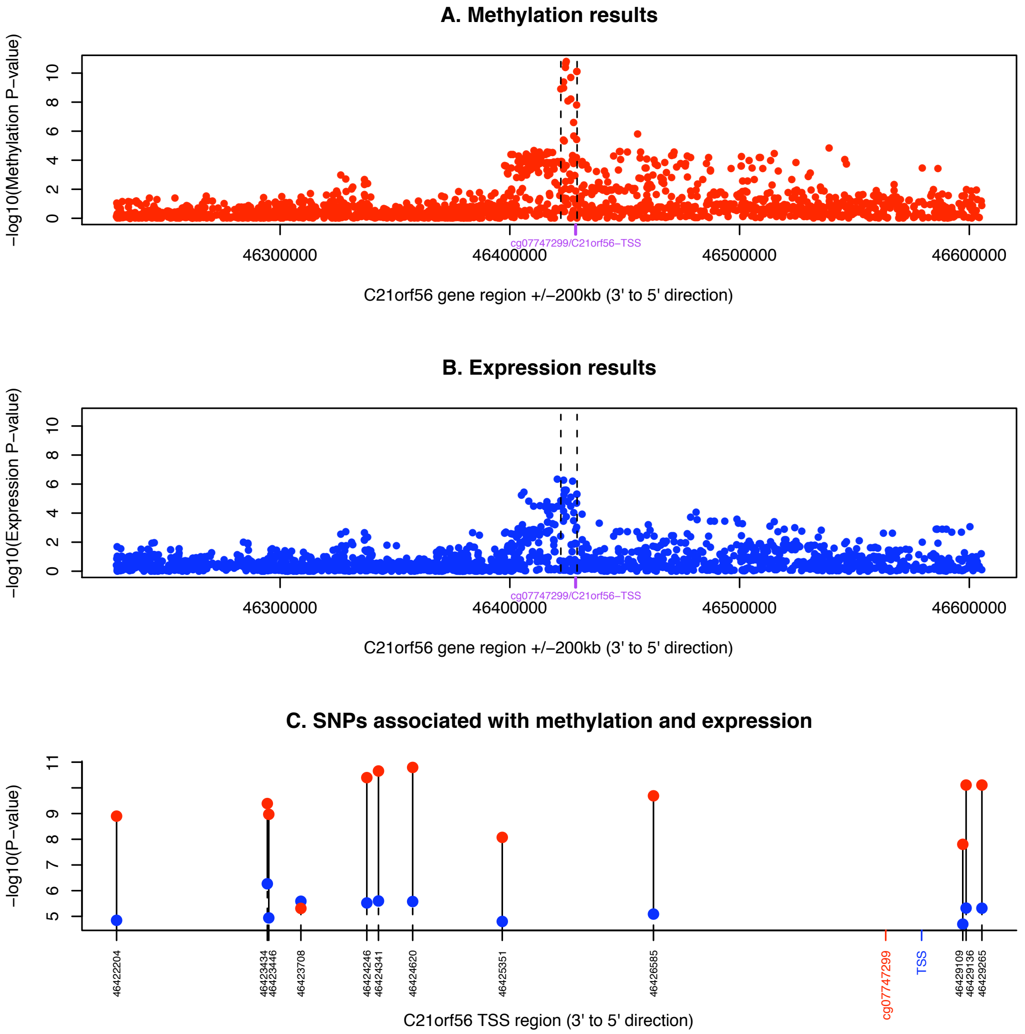


Figure S11. Association results for the *C21orf56* gene region with SNP genotype data from 1000 Genomes Project. Genotypes were available for 44 individuals in our sample and missing data were imputed using the posterior mean genotype in BIMBAM [56]. (A) Methylation association results for 76 individuals for methylation probe cg07747299. (B) Gene-expression association results for 69 individuals for *C21orf56*. Dashed lines indicate the region in panel C. (C) Twelve SNPs with significant (FDR 10%) evidence for association to both methylation (red) and gene-expression (blue).

# 6 Supplementary Tables

Table S1. Top SNP association results at FDR of 10% ($P < 10^{-7}$) for the variation in overall autosomal methylation means and principal components. For each result we present the most-associated SNP from all SNPs within 1Mb of each other and the nominal P-value.

| Rank | Chromosome | SNP | Location | PC | P-value |
|---|---|---|---|---|---|
| 1 | 12 | rs10876043 | 49190411 | 1 | $4.51 \times 10^{-9}$ |
| 2 | 2 | rs12619100 | 53703886 | 73 | $7.73 \times 10^{-9}$ |
| 3 | 4 | rs12330972 | 136212036 | 61 | $1.04 \times 10^{-8}$ |
| 4 | 13 | rs496313 | 108615052 | 1 | $2.34 \times 10^{-8}$ |
| 5 | 1 | rs6429144 | 237836763 | 59 | $3.25 \times 10^{-8}$ |
| 6 | 10 | rs1777331 | 29957509 | 64 | $3.32 \times 10^{-8}$ |
| 7 | 6 | rs6571070 | 103487663 | 45 | $4.20 \times 10^{-8}$ |
| 8 | 3 | rs5007165 | 152097786 | 15 | $4.42 \times 10^{-8}$ |
| 9 | 14 | rs17256246 | 22936433 | 70 | $4.67 \times 10^{-8}$ |
| 10 | 4 | rs7665432 | 37056958 | 17 | $5.00 \times 10^{-8}$ |
| 11 | 14 | rs1272916 | 62496523 | 13 | $5.59 \times 10^{-8}$ |
| 12 | 2 | rs1158867 | 127893847 | 4 | $7.61 \times 10^{-8}$ |

Table S2. 37 probes with evidence for 44 *cis* and *trans* associations. For each probe we present the most-associated SNP from all SNPs within 1Mb and the nearest gene.

| Rank | CG-chr | CG-bp | CG | SNP-chr | SNP-bp | SNP | Gene |
|---|---|---|---|---|---|---|---|
| 1 | 21 | 37284290 | cg27519424 | 21 | 37286855 | rs2187102 | HLCS |
| 2 | 19 | 9800038 | cg17704839 | 17 | 27579063 | rs7225527 | UBL5 |
| 3 | 1 | 158161630 | cg08661227 | 1 | 158161583 | rs1317846 | TAGLN2 |
| 4 | 11 | 64838696 | cg01630869 | 11 | 64845074 | rs4149835 | CDC42EP2 |
| 5 | 1 | 229182620 | cg10324224 | 1 | 229182510 | rs10864645 | ARV1 |
| 6 | 4 | 114342 | cg12717203 | 4 | 157596 | rs10027536 | ENSG00000211553 |
| 7 | 3 | 151963600 | cg24856383 | 3 | 151962747 | rs1444197 | SIAH2 |
| 8 | 19 | 18493488 | cg02344201 | 19 | 18460792 | rs2013071 | ELL |
| 9 | 4 | 39205737 | cg09067967 | 1 | 200731383 | rs3817222 | UGDH |
| 10 | 4 | 39205737 | cg09067967 | 1 | 143702635 | rs1778596 | UGDH |
| 11 | 16 | 21927177 | cg10052190 | 4 | 7998166 | rs2891926 | C16orf52 |
| 12 | 7 | 31693179 | cg23216015 | 7 | 31694705 | rs12666349 | C7orf16 |
| 13 | 17 | 36451247 | cg02022375 | 17 | 36439391 | rs16968916 | ENSG00000188581 |
| 14 | 1 | 58815787 | cg16080552 | 1 | 58744419 | rs3087585 | TACSTD2 |
| 15 | 5 | 60275337 | cg10506318 | 5 | 60269593 | rs2694518 | ERCC8 |
| 16 | 15 | 72952949 | cg10253484 | 15 | 72992726 | rs12439641 | SCAMP2 |
| 17 | 4 | 39205737 | cg09067967 | 5 | 40273131 | rs36019094 | UGDH |
| 18 | 6 | 166716734 | cg17391474 | 6 | 166718512 | rs7449629 | BRP44L |
| 19 | 4 | 114693 | cg15792688 | 4 | 157596 | rs10027536 | ENSG00000211553 |
| 20 | 8 | 124264314 | cg23067535 | 8 | 124264215 | rs16898097 | FAM83A |
| 21 | 4 | 39205737 | cg09067967 | 3 | 16435774 | rs17042252 | UGDH |
| 22 | 17 | 46199123 | cg03016571 | 17 | 46193310 | rs9910392 | C17orf73 |
| 23 | 19 | 60274447 | cg27105123 | 19 | 60276005 | rs1654468 | EPS8L1 |
| 24 | 4 | 39205737 | cg09067967 | 10 | 120886446 | rs34566675 | UGDH |
| 25 | 4 | 39205737 | cg09067967 | 7 | 63937080 | rs6944297 | UGDH |
| 26 | 1 | 39320114 | cg13514129 | 1 | 244476735 | rs1361409 | MACF1 |
| 27 | 5 | 81303384 | cg06777715 | 4 | 1283302 | rs6837701 | ATG10 |
| 28 | 12 | 6904172 | cg01437411 | 12 | 6903853 | rs7310941 | ATN1 |
| 29 | 1 | 47262126 | cg16689634 | 1 | 160147106 | rs12079579 | CYP4X1 |
| 30 | 7 | 129720250 | cg01796223 | 7 | 129720717 | rs10954267 | CPA4 |
| 31 | 21 | 46428480 | cg07747299 | 21 | 46424341 | rs8133082 | C21orf56 |
| 32 | 19 | 15869723 | cg13656062 | 19 | 15869512 | rs3093098 | CYP4F2 |
| 33 | 11 | 33018483 | cg18495563 | 11 | 33025419 | rs12794247 | TCP11L1 |
| 34 | 17 | 44161045 | cg21842478 | 12 | 65529862 | rs1504307 | HOXB13 |
| 35 | 9 | 6005507 | cg18906795 | 12 | 31224049 | rs2638971 | RANBP6 |

Table S2 Continued.

| Rank | CG-chr | CG-bp | CG | SNP-chr | SNP-bp | SNP | Gene |
|---|---|---|---|---|---|---|---|
| 36 | 4 | 88562960 | cg21298523 | 4 | 88557459 | rs12640451 | NUDT9 |
| 37 | 3 | 51870732 | cg14940420 | 3 | 51874308 | rs9875459 | IQCF2 |
| 38 | 11 | 43658971 | cg11948328 | 6 | 157707014 | rs9459183 | HSD17B12 |
| 39 | 14 | 64072269 | cg25797366 | 14 | 64073644 | rs7151976 | HSPA2 |
| 40 | 17 | 7152009 | cg24181591 | 17 | 7156534 | rs8075575 | ENSG00000132507 |
| 41 | 9 | 6005507 | cg18906795 | 12 | 9510972 | rs17804971 | RANBP6 |
| 42 | 6 | 136613336 | cg17754980 | 6 | 150772148 | rs616195 | FAM54A |
| 43 | 4 | 39205737 | cg09067967 | 15 | 82613080 | rs17301021 | UGDH |
| 44 | 3 | 73128376 | cg20540428 | 3 | 73128341 | rs9816164 | PPP4R2 |

Table S3. SNPs of interest included in Figure S8. These include all SNPs within 200kb of 14 candidate genes and previously associated (not always corrected for multiple testing) SNPs [15, 16, 18, 5, 17, 7, 66, 8].

| Chromosome | Start | End | SNP(s) | Source |
|---|---|---|---|---|
| 1 | 8856709 | 8446028 | 267 | AID |
| 1 | 11988702 | 11568373 | 360 | MTHFR |
| 1 | 32926044 | 32503244 | 196 | ZBTB8B |
| 1 | 33044129 | 32577358 | 230 | ZBTB8A |
| 2 | 25618278 | 25109348 | 289 | DNMT3A |
| 3 | 130841542 | 130432482 | 307 | MBD4 |
| 9 | 6697051 | 6203150 | 489 | UHRF2 |
| 17 | 7528292 | 7103408 | 311 | ZBTB4 |
| 18 | 46262142 | 45849213 | 574 | MBD1 |
| 18 | 50205156 | 49734572 | 610 | MBD2 |
| 19 | 5113165 | 4660509 | 328 | UHRF1 |
| 19 | 10366755 | 9905022 | 300 | DNMT1 |
| 20 | 31060823 | 30613851 | 346 | DNMT3B |
| 21 | 44706527 | 44290650 | 359 | DNMT3L |
| NA | NA | NA | 16 | Kerkel et al. Suppl. Table 1 |
| NA | NA | NA | 21 | Schalkwyk et al. Table 1 |
| NA | NA | NA | 10 | Zhang et al. Table 1 |
| NA | NA | NA | 7 | Kadota et al. Figure 4. |
| NA | NA | NA | rs2334499 | Kong et al. |
| NA | NA | NA | 106 | Boks et al. Suppl. Table 4 |
| NA | NA | NA | 25999 | Gibbs et al. Suppl. Table 3 |

Table S4. Eighteen genes with previous evidence for differential methylation or expression. Pubmed search terms included the gene name and ”methylation” or ”imprinting” in the title or abstract text. We present genes with evidence for differential methylation (DMR), that could also show differential gene-expression (DER) and correlations between gene-expression and methylation (Methylation-expression).

| Gene | Pubmed hits | DMR | DER | Methylation-expression |
|---|---|---|---|---|
| HSD17B1 | 1 | y | n | n |
| IRF7 | 8 | y | n | n |
| MAP2K3 | 2 | y | n | n |
| MAPK13 | 2 | y | y | y |
| KLK10 | 4 | y | y | y |
| S100P | 5 | y | y | y |
| PAX6 | 18 | y | n | n |
| TRPM2 | 1 | y | y | y |
| CDKN1A | 14 | y | n | n |
| HSPA2 | 1 | y | n | n |
| KLF11 | 1 | y | n | n |
| MUC4 | 4 | y | n | n |
| LARS2 | 1 | y | y | y |
| PERP | 1 | y | y | y |
| CD3G | 1 | y | n | n |
| HLCS | 1 | y | n | n |
| SLC10A1 | 1 | y | y | y |
| SYK | 28 | y | y | y |
